# Exploring the size limits of Bionano optical genome mapping to resolve alternative structures of linked interspersed chromosomal duplications

**DOI:** 10.1186/s13073-025-01571-0

**Published:** 2025-11-13

**Authors:** Yang Pei, Eduardo Calpena, Jill M. Brown, Ron Schwessinger, Lucy Platts, Simon J. McGowan, Tazeen Ashraf, Francesca Forzano, Jane A. Hurst, Wendy D. Jones, Ajoy Sarkar, Richard J. Gibbons, Stephen R. F. Twigg, Andrew O. M. Wilkie

**Affiliations:** 1 Clinical Genetics Group, MRC Weatherall Institute of Molecular Medicine, University of Oxford, John Radcliffe Hospital, Oxford, OX3 9DS UK; 2 NIHR Oxford Biomedical Research Centre, Oxford, OX3 9DU UK; 3 Grupo de Investigación en Biomedicina Molecular, Celular y Genómica, Unidad CIBERER (U755), Instituto de Investigación Sanitaria La Fe (IIS La Fe), Valencia, Spain; 4 MRC Molecular Haematology Unit, MRC Weatherall Institute of Molecular Medicine, University of Oxford, John Radcliffe Hospital, Oxford, OX3 9DS UK; 5 Rare and Inherited Disease Laboratory, NHS North Thames Genomic Laboratory Hub, Great Ormond Street Hospital for Children NHS Foundation Trust, Great Ormond Street Hospital, London, WC1N 3JH UK; 6 Centre for Computational Biology, MRC Weatherall Institute of Molecular Medicine, University of Oxford, John Radcliffe Hospital, Oxford, OX3 9DS UK; 7 North East Thames Regional Genetics Service, Great Ormond Street Hospital for Children NHS Foundation Trust, Great Ormond Street Hospital, London, WC1N 3JH UK; 8 Clinical Genetics Department, Thomas’ NHS Foundation Trust, Guy’s Hospital, Guy’s & St, London, SE1 9RT UK; 9 Faculty of Life Sciences & Medicine, King’s College London, Guy’s Campus Great Maze Pond, London, SE1 1UL UK; 10 Clinical Genetics Service, Nottingham University Hospitals NHS Foundation Trust, City Hospital, Nottingham, NG5 1PB UK

**Keywords:** Optical genome mapping, Chromosomal duplication, Triplication, Translocation, Hypertrichosis, Gingival hyperplasia, HTC3, *KCNJ2*, Topologically-associating domain, Craniosynostosis, Chromoanasynthesis

## Abstract

**Background:**

Determining the correct structure of large, interspersed duplications and related complex genomic rearrangements in genetic disease is critical when establishing causal roles and requires a technology able to span the entire duplicated segment(s) on single molecules. We assessed the use of Bionano optical genome mapping (OGM) for this purpose.

**Methods:**

We combined OGM, Illumina short-read sequencing and fluorescence in situ hybridisation (FISH) to characterise three large interspersed duplications/triplications, and used the deepC algorithm to predict impact on local topologically-associating domains (TADs), assisting functional interpretation.

**Results:**

Case 1 harboured paired interspersed duplications (244/323 kb) on chromosome 13. By analysing multiple molecules > 300 kb completely spanning the smaller duplication, we unambiguously determined the correct structure, which potentially alters the TAD containing *FGF9*, a candidate gene. In Case 2, involving a child with hypertrichosis and gingival hyperplasia (HTC), duplications on chromosomes 16 (2.01 Mb) and 17 (564 kb) were linked on short-read sequencing. By obtaining three OGM molecules spanning the 564 kb segment, we deduced that a t(16;17) translocation was present, which we confirmed by FISH. This interpretation has important implications for clinical risk and highlights *KCNJ2* as a potential driver of the *HTC3* locus at 17q24.3. Case 3 involved a complex chromoanasynthesis event on chromosome 20. OGM readily resolved all but two of 12 alternative structures; however full resolution required reads to span a 627 kb duplication, which we could not achieve consistently.

**Conclusions:**

OGM represents a powerful tool for disambiguating complex structural variants, but requires multiple individual reads to completely span the duplicated segment. In our hands the upper size of duplications that could be resolved was ~ 550 kb. Deducing the correct configuration is critical both for mechanistic understanding of pathogenesis and accurate recurrence risk counselling.

**Supplementary Information:**

The online version contains supplementary material available at 10.1186/s13073-025-01571-0.

## Background

Following the introduction of array diagnostics into clinical use around fifteen years ago, the frequent occurrence of copy number variants (CNVs) below the limits of standard karyotypic resolution (2–5 Mb) was evident. Once it became possible to relate this information to the genome sequence of the corresponding regions, unprecedented structural complexity was uncovered. This was originally documented in both constitutional [1] and somatic [2] aberrations associated with disease states, but more recently has been catalogued and classified in healthy individuals. For example, Collins et al. [3] classified complex structural variants (cxSVs) (defined as “non-canonical rearrangements involving two or more distinct SV signatures or at least three linked breakpoints”) into 16 different classes. In one relatively frequent class termed “DUP-INV-DUP”, pairs of duplicated (DUP) segments flank a single copy region that is inverted (INV), usually together with part or all of the duplicated segment [4].

**Fig. 1:**
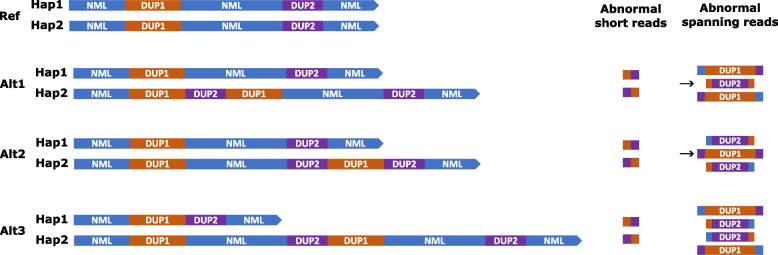
Linked interspersed duplications are compatible with multiple alternative structures. The topmost cartoon (Ref) shows the normal configuration of a section of an autosomal chromosome pair. In the variant individual shown below, two interspersed segments (DUP1 [orange] and DUP2 [purple]) have been duplicated: short-read sequencing of the boundaries of the duplicated regions reveals two abnormal breakpoints indicating that DUP1 and DUP2 are mutually linked together (shown diagrammatically in column “Abnormal short reads”). Alt1, Alt2 and Alt3 are three alternative structures for these linked interspersed duplications, which are indistinguishable based only on copy number and breakpoint sequences; note that Alt3 involves a translocation between the homologous chromosomes. NML indicates the intervening and flanking blue sequences, which are present in normal copy number, hence the reference sequence has structure NML-DUP1-NML-DUP2-NML. The column “Abnormal spanning reads” shows how long reads that completely span one of the duplicated segments can be used to distinguish the alternative structures based on the flanking sequences on either side. However, only the reads indicated with arrows are uniquely diagnostic of a particular structure (Alt1 or Alt2); no reads are uniquely diagnostic of Alt3, hence FISH is the preferred technology to confirm translocations

In a variation of this rearrangement, two or more pairs of duplications, oriented in the same direction, are sourced from sequences on the same chromosome that are separated by a normal copy-neutral (NML) region (“DUP-NML-DUP”) and are physically linked together. In its simplest manifestation three alternative outcomes from such events are possible (Fig. 1), in two of which (Alt1 and Alt2) different structures are created on the same chromosome (which we term “linked interspersed duplications”), whereas the third (Alt3) requires a translocation to have occurred between the homologous chromosomes. In constitutional disease such DUP-NML-DUP events, although rare, have been described in several case series [5–9]. However as shown in Fig. 1 and noted in several reports, the true structure of these rearrangements is often ambiguous [7–13]. Resolving the correct structure can be time-consuming and resource-intensive, but may be critical for establishing the pathogenicity of the rearrangement. For example in a recent report [14], duplications of 92 kb and 91 kb on chromosome 6 were separated by 2.1 Mb of copy-neutral sequence. One form of the rearrangement was predicted to introduce a pathogenic insertion into the *ENPP1* gene, whereas the other would leave *ENPP1* intact. In the event, *ENPP1* was shown to be disrupted and this was explanatory of the disease in the patient [14].

In formulating a general approach to resolving the structure of such rearrangements, a key concept is that the technology must be able to *visualise simultaneously the sequence lying on either side of the duplicated segment in single molecules* (see “Abnormal spanning reads” in Fig. 1). The most appropriate technology to use will range from long PCR, for duplications spanning under 10 kb, to fluorescence in situ hybridisation (FISH) for megabase-scale duplications. One technology that can potentially fill the gap between these extremes is optical genome mapping (OGM), in which patterns of labels on intact DNA molecules are directly visualised and compared to a reference, to detect structural variation. Implemented on the Bionano platform, this has been successful in solving many constitutional and somatic rearrangements [13, 15–19]. Current Bionano company literature states that the DNA isolation protocol routinely yields average fragments > 230 kb (https://bionano.com/sample-preparation-kits/, accessed 25 July 2025); correspondingly, the *ENPP1* rearrangement mentioned above (comprising duplications of 91 kb and 92 kb) was readily resolved using this technology [14].

As part of a project to investigate the pathological role of cryptic CNVs in craniosynostosis (premature fusion of one or more sutures of the skull vault [20]), we used Bionano OGM to explore the genomes of 20 unrelated families with this condition [21, 22]. Here we describe the investigation (in order of increasing difficulty) of three patients from this series who harboured cxSVs, including a total of seven larger (> 200 kb) duplications ranging in size from 205 kb – 2.01 Mb. In addition to causing direct disruption of gene function (as in the *ENPP1* example above), we also considered the alternative possibility that large cxSVs could impact gene regulation through perturbation of the normal conformation of topologically-associating domains (TADs) [23]. This work illustrates some of the challenges in characterising such rearrangements and the strengths and weaknesses of OGM in their investigation.

## Methods

### Ethics

The clinical studies were approved by the relevant Research Ethics Committees (REC), as described in the Ethics Declaration (see later section). Written informed consent was obtained for each participating individual.

### Cohort

Whole blood was obtained for DNA preparation from three probands (Cases 1–3) with a confirmed diagnosis of craniosynostosis, together with their unaffected parents; the unaffected maternal grandmother of Case 3 was also included. The probands originated from two cohorts of patients with multi-suture or syndromic craniosynostosis recruited in England (United Kingdom); each patient had previously been analysed by clinical sequencing of diagnostic genes and array comparative genomic hybridisation (aCGH), followed by research-based exome and/or genome sequencing, without a definitive genetic diagnosis having been obtained [24, 25]. Cases 1 and 3 were sourced from a re-analysis of genome sequencing data (*n* = 114 unrelated cases) from the 100,000 Genomes Project (100kGP) by Hyder et al. [25]. Case 2 was sourced from patients (*n* = 40 unrelated cases) recruited to a study of exome/genome sequencing in craniosynostosis by Miller et al. [24]; we performed additional genome sequencing of the proband to enable exact molecular characterisation of chromosomal rearrangements previously detected by aCGH. Subsequently, the three cases were analysed by Bionano OGM as part of a wider survey of OGM in craniosynostosis [21, 22]. This revealed that each independently harboured large (> 200 kb) linked interspersed duplications, leading to the option to present these three cases as an illustrative series.

### Array comparative genomic hybridisation (aCGH) and FISH

aCGH studies were performed by the respective clinical laboratories investigating the patient. Researchers had access to the clinical reports but not the primary data. FISH was carried out using standard procedures [26] on cells obtained from an induced pluripotent stem cell (iPSC) line derived from whole blood (Case 1) (Korona D, Hashimoto AS, Pei Y, Calpena E, Sloane-Stanley J, Riva SG, Schwessinger R, Forzano F, Chintawar S, Duggal G, Wall SA, Hughes JR, Twigg SRF, Wilkie AOM: Evaluating the pathogenic significance of unique chromosomal variants in craniosynostosis using patient-derived induced pluripotent stem cells and mouse modelling, submitted) or by a clinical laboratory on slides prepared from a fresh blood sample (Case 2). The FISH probes and fluorophores used in each analysis are provided in Additional file 1: Table S1.

### Exome and genome sequencing

Exome sequence data on the trios from Cases 2 and 3 were generated as part of the study reported by Miller et al. [24]. Genome sequence data on the trios from Cases 1 and 3 were generated as part of the 100kGP as described in Smedley et al. [27]. Data (Data Release V7, 25/07/19) were accessed in the Genomics England Research Environment (RE) as part of the research study Molecular Genetics of Craniosynostosis (Research Registry project 65); approval was obtained for export of all data and figures provided in this work. Methods for SV detection in Manta [28] and Canvas [29] files provided for each sequence in the RE were reported by Hyder et al. [25] in the Supplementary Information section ("Structural and copy number variants") of that publication. Genome sequence data [30] on the trio from Case 2 were generated by Novogene according to the NovaSeq PE150 sequencing strategy (Illumina NovaSeq 6000), aligned with bwa-mem [31] and processed with Samtools v1.1. Except where stated, all genomic coordinates use the GRCh38 reference. SV visualisation was carried out with Samplot [32], while the sequencing depth information for the coverage plot (Additional file 1: Fig. S13B) was extracted directly from the bam files using Samtools. However, Case 1 was originally analysed in GRCh37/hg19 within the RE, therefore Additional file 1: Table S6 and Fig. S1 are provided in that reference build. In addition, all deepC predictions (described below) were undertaken in hg19. For each CNV, the conversion between GRCh37/hg19 and GRCh38 reference is provided in Additional file 1: Table S2.

### Breakpoint PCR

All abnormal breakpoints predicted from analysis of the genome sequence were confirmed by breakpoint PCR and dideoxy-sequencing of DNA from the appropriate individuals. The primers used for each breakpoint PCR are provided in Additional file 1: Table S3. Breakpoint PCR was performed using the Bio-Rad T100™ Thermal Cycler and FastStart Taq DNA Polymerase (Roche, FTAQ-RO) following a modified FastStart Taq PCR protocol. Briefly, 1 μL genomic DNA (20 μg/μL) was amplified in 20 μL reactions with 10 mM dNTPs (Roche, 04738403001), 10 × Standard Taq Reaction Buffer (Roche, 12161567001), and 10 μM of primers, which are listed in Additional file 1: Table S3. Thermal-cycling conditions were as follows: 95 °C for 8 min; followed by 32 cycles of 95 °C for 30 s, 60 °C for 30 s and 72 °C for 1 min/kb; followed by 72 °C for 10 min. PCR products were electrophoresed on 2% agarose gels.

### In silico prediction of topologically-associating domains (TADs)

TAD prediction was carried out with deepC [33], where the model was trained on the IMR-90 lung fibroblast data from Rao et al. [34] using the hg19 reference, excluding chromosomes 12, 13, 16 and 17 from training. Input sequence for each variant was generated by reconstructing the respective reference sequence based on the precise breakpoint coordinates determined by breakpoint PCR and dideoxy-sequencing.

### Optical genome mapping (OGM) using the Bionano platform

High molecular weight DNA for OGM was extracted from lymphoblastoid cell pellets (Case 1) or snap-frozen blood (Cases 2 and 3) using the “SP Blood & Cell Culture DNA Isolation Kit v2, product number: 80042” following the “Bionano Prep SP Frozen Human Blood DNA Isolation Protocol, 30246, revision F” protocol for blood and the “Bionano Prep SP Frozen Cell Pellet DNA Isolation Protocol v2 - 30398, revision B” for cell pellets. Extracted DNA was processed for OGM using the “Direct Label and Stain (DLS) Kit, part number 80005” following the “Bionano Prep Direct Label and Stain (DLS) Protocol, 30206, revision G”. To collect OGM data, the DLS DNA was loaded into the Saphyr chip (version G1.2, two samples per chip) and mounted into the Saphyr machine, following the “Saphyr® System User Guide, Document number 30143, revision C”. The maximum amount of data (1.5 Tb) was collected when possible.

All subsequent analyses of the OGM data [30] were performed on the Bionano Access platform (version 1.6.1) with the Bionano Solve software (version 3.6.1_11162020). Data from each OGM run were subjected to quality control (QC) to ensure run metrics met the recommended threshold according to the Bionano “Data Collection Guidelines, Document Number: 30173, Revision: E”. Quality control metrics for each sample analysis contributing to this work are provided in Additional file 1: Table S4; all samples met minimum quality standards.

De novo assembly analysis was performed on unfiltered molecules and after down-sampling by retaining molecules larger than > 300 kb (Cases 1 & 2) and > 320 kb (Case 3). Rare Variant Analysis (RVA) was performed additionally for Cases 2 and 3 and a maximum 1.5 Tb data were available for both. The requirements for an individual molecule to be considered informative towards determining structure were: (1) each side flanking the duplicated segment of interest included a minimum of 4 labels that distinctively matched either the expected NML pattern or the anticipated linked DUP pattern; and (2) the NML pattern was observed in only one, or neither, of these flanking regions (Fig. 1). After the correct structure was determined, the pathogenicity of each cxSV was scored according to American College of Medical Genetics (ACMG) criteria, supplemented by additional guidance for non-coding regions (Additional file 1: Table S5) [35–37].

## Results

### Case 1: duplications of 244 kb and 323 kb on chromosome 13

The proband presented with sporadic syndromic sagittal craniosynostosis and developmental delay, whilst her parents are not similarly affected (see Additional file 1, Case Descriptions and Analysis, Case 1). Routine clinical workup included aCGH, which was reported as showing only a paternally inherited duplication on chromosome 16, considered likely to be coincidental to the phenotype. The family was referred to the 100kGP for genome sequencing of the parent–child trio. Using methods reported by Hyder et al. [25], systematic interrogation of the Manta [28] and Canvas [29] datasets identified two duplicated segments on chromosome 13 (Additional file 1: Table S6). Both duplications had arisen de novo as they were not evident in either parental sample, based on Samplot analysis of read copy number (Additional file 1: Fig. S1). Detailed scrutiny of the sequences at the breakpoints using the Integrative Genomics Viewer (IGV) [38] revealed three duplicated segments, two large (244 kb and 323 kb) and a third of just 148 bp that was inverted in the duplicated copy, all located within the 13q12.11-q12.12 region. The sequence relationships between the three duplicated segments, deduced from IGV, were confirmed by breakpoint PCR and dideoxy-sequencing (Additional file 1: Fig. S2). The extent of sequence homology at each breakpoint is summarised in Table 1. Based on analysis of common single nucleotide variants (SNVs) and indels, both large duplications were of paternal origin; the two copies of the 323 kb duplication originated from heterologous paternal chromosomes, whereas the 244 kb copies appeared identical, indicating templating of the duplicated copy by sequence from the same paternal chromosome (Additional file 1: Fig. S3).

**Table 1:** Metrics for 10 interspersed duplications and associated abnormal break junctions

DUP ID	Chromosome	DUP Start	DUP End	Size (bp)	Copy number	DUP chromosomal origin	Break junction (:) and strand orientation (+/-)	Homology (bp)	Proposed mechanism^a^
13.1	13	22,222,612	22,545,849	323,238	2	Heterologous	+ 13.1:−13.2	5	MMBIR
13.2	13	23,312,551	23,312,698	148	2	Heterologous	−13.2: + 13.3	0	NHEJ
13.3	13	23,609,688	23,854,091	244,404	2	Homologous	+ 13.3: + 13.1	23	Alu-Alu homology
16	16	77,258,535	79,267,342	2,008,808	2	Homologous	+ 16: + 17	2	MMBIR
17	17	69,913,550	70,477,256	563,707	2	Homologous	+ 17: + 16	0	TINS (6 nucleotide inversion)
20.1	20	9,153,480	9,780,418	626,939	2	Heterologous	−20.1:−20.5	38	LTR homology
20.2	20	12,115,100	12,319,713	204,614	2	Heterologous	+ 20.2:−20.1	4	MMBIR
20.3	20	12,350,710	12,564,055	213,346	2	?Homologous	−20.3:−20.4	0	NHEJ
20.4	20	12,564,056	12,626,497	62,442	3	Heterologous	−20.4: + 20.2	2	MMBIR
20.5	20	12,626,498	12,657,715	31,218	2	?Homologous			

^a^LTR, long terminal repeat; MMBIR, microhomology-mediated break-induced repair; NHEJ, non-homologous end-joining; TINS, templated insertion

**Fig. 2:**
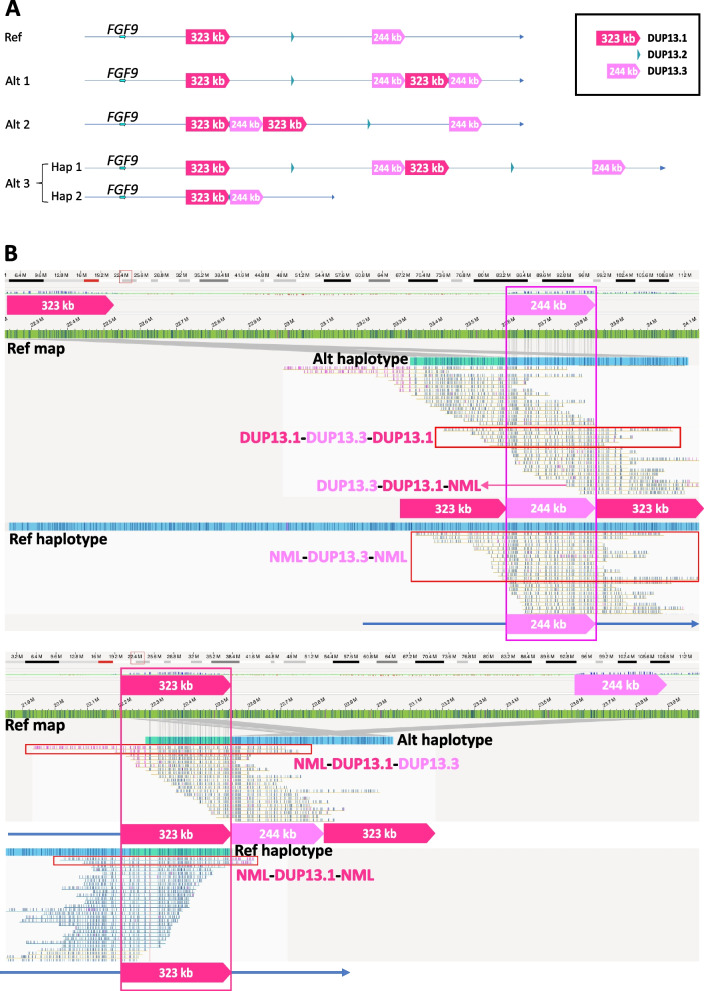
Use of Bionano OGM to distinguish three alternative configurations for the Case 1 cxSV. **A** Diagram illustrating three different configurations Alt1, Alt2, and Alt3 (the latter involving both chromosome 13 homologues), all of which are compatible with the breakpoint sequence and copy number data shown in Additional file 1: Fig. S1 and Fig. S2. Inset shows numbering terminology of individual duplicated (DUP) segments (13.1, 13.2 13.3) as summarised in Table 1. **B** Alignment of individual OGM reads, including reads completely spanning one or other of the large duplicated regions (vertical pink boxes), and comparison with reference maps; NML indicates regions not subject to duplication. Importantly, five molecules spanning the 244 kb segment (DUP13.3) have flanking labelling patterns diagnostic of the 323 kb segment (DUP13.1) on either side (top red box; DUP13.1-DUP13.3-DUP13.1). The remaining molecules spanning the 244 kb segment all have normal flanking banding patterns (second red box from top; NML-DUP13.3-NML). Note that the DUP13.1-DUP13.3-DUP13.1 pattern is unique to Alt2, as shown in part A. Also detected were a smaller number of abnormal molecules spanning the larger 323 kb segment. Two types are seen, a single DUP13.3-DUP13.1-NML (pink arrow) and two NML-DUP13.1-DUP13.3 (enclosed by third red box from top); both types are consistent with Alt2, but are also present in Alt3, so not diagnostic on their own. Note that the small (148 bp) DUP13.2 is not included in this analysis, because it is not resolvable by OGM

Whilst the analysis described so far provided a nucleotide level characterisation of the rearrangement, it did not distinguish the three different possible topological relationships (denoted Alt1, Alt2, Alt3) of the duplicated segments, as illustrated in Fig. 2A (see inset for numbering terminology of the segments). Distinguishing these possibilities could be important for functional interpretation: for example, in Alt2 and Alt3, a copy of the 244 kb segment is brought into closer proximity with *FGF9*, a known disease gene in craniosynostosis [39, 40], whereas in Alt1, the rearrangement occurs remotely to this gene. We asked whether Bionano OGM could distinguish these alternative topologies.

In our initial exploration of this question, we followed the automated de novo assembly pipeline using the Bionano Access software provided with the Saphyr instrument. This produced a confusing result seemingly compatible with either Alt1 or Alt3 (Additional file 1: Fig. S4), probably generated by the pileup of molecules that did not fully span the duplicated segments, leading to false haplotype switching. After down-sampling and only including long and therefore informative molecules (that is, fully spanning a duplicated segment), the de novo analysis reached a convincing solution, shown in Fig. 2B. Specifically, five molecules that completely span the smaller 244 kb DUP clearly exhibited the signature of the larger 323 kb DUP on both sides (DUP13.1-DUP13.3-DUP13.1), a structure only compatible with Alt2. All other molecules spanning the 244 kb DUP had a normal flanking sequence context on both sides, a result also only compatible with Alt2 (Fig. 2B). In this experiment, only four molecules spanned the larger 323 kb segment, three with NML-DUP13.1-DUP13.3 topology and one with DUP13.3-DUP13.1-NML topology (Table 2); although these results are compatible with Alt2, it should be noted that the Alt2 and Alt3 configurations are indistinguishable based on analysis of the 323 kb DUP-spanning molecules (Fig. 2A). We used 3-colour FISH to check that the Alt2 configuration deduced by OGM was correct. This yielded multiple cells with a sequence of labels characteristic of Alt 2 (Additional file 1: Fig. S5), supporting the OGM result.

**Table 2:** Number of completely spanning molecules supporting each abnormal DUP topology present in the proband samples, arranged by DUP size

Abnormal DUP topology	DUP size (bp)	Number of molecules
13.1–13.2–13.3	148	[0]^a^
20.1–20.5-NML	31,218	> 121
20.3–20.4–20.2	62,442	140
NML-20.2–20.1	204,614	34
20.4–20.2-NML	204,614	38
NML-20.3–20.4	213,346	53
13.1–13.3–13.1	244,404	5
NML-13.1–13.3	323,238	3
13.3–13.1-NML	323,238	1
16–17-NML	563,707	3
NML-17–16	563,707	0
20.2–20.1–20.5	626,939	0^b^
NML-16–17	2,008,808	0
17–16-NML	2,008,808	0

^a^This DUP is too small for OGM to resolve

^b^A single spanning molecule was identified in the mother of Case 3 (Fig. 4B)

To assess the potential functional significance of the Alt2 rearrangement, we used deepC [33] to assist in prediction of effects on local TAD structure. Interestingly, the candidate gene *FGF9*, previously implicated in craniosynostosis [39, 40], lies at one end of a large (1.65 Mb) TAD identified in IMR-90 lung fibroblasts [34], that fully includes the native 323 kb region (Additional file 1: Fig. S6). The Alt2 rearrangement would introduce additional copies of the 244 kb and 323 kb DUP segments within this large TAD, and deepC predicts that Alt2 may alter the internal interactions of the TAD; of particular note, the newly inserted 244 kb segment is annotated as harboring part of a craniofacial superenhancer (Additional file 1: Fig. S6, lower panel) [41]. Hence, these observations could be compatible with a regulatory effect of the Alt2 rearrangement on *FGF9* expression.

As described in a separate investigation (Korona D, Hashimoto AS, Pei Y, Calpena E, Sloane-Stanley J, Riva SG, Schwessinger R, Forzano F, Chintawar S, Duggal G, Wall SA, Hughes JR, Twigg SRF, Wilkie AOM: Evaluating the pathogenic significance of unique chromosomal variants in craniosynostosis using patient-derived induced pluripotent stem cells and mouse modelling, submitted), we generated iPSCs from the affected individual, differentiated these to a neural crest identity, and used RNA sequencing and quantitative reverse transcriptase-PCR to measure alterations in expression of genes in a ± 2 Mb window around the rearrangement. Notably, *FGF9* showed the greatest fold-upregulation (2.7x) of 65 transcripts within the region, consistent with the TAD modelling and supporting a likely causal role of the rearrangement for the craniosynostosis phenotype (Korona D, Hashimoto AS, Pei Y, Calpena E, Sloane-Stanley J, Riva SG, Schwessinger R, Forzano F, Chintawar S, Duggal G, Wall SA, Hughes JR, Twigg SRF, Wilkie AOM: Evaluating the pathogenic significance of unique chromosomal variants in craniosynostosis using patient-derived induced pluripotent stem cells and mouse modelling, submitted).

Applying dosage criteria alone [35] the chromosome 13 rearrangement was classified as a variant of uncertain significance (VUS); however by adding the functional evidence and following suggested adaptations to ACMG criteria for non-coding regions [36, 37], it was re-classified as likely pathogenic (Additional file 1: Table S5). Complicating the interpretation however, Case 1 also harbours a pathogenic de novo single nucleotide deletion in the *FOXP2* gene, providing a competing explanation for the child’s clinical features (see Additional file 1 for further details).

### Case 2: duplications of 2.01 Mb on chromosome 16 and 564 kb on chromosome 17

Case 2 is a sporadically affected individual who presented with a combination of multisuture craniosynostosis, marked hypertrophy of the gums, dental disruption and hairy external auditory meati (see Additional file 1, Case Descriptions and Analysis, Case 2). Array CGH demonstrated duplications on 16q23.1 (shown to have arisen de novo) and 17q24.3 (parents not investigated), which were both classed as being of uncertain clinical significance. To characterise these duplications molecularly, we undertook genome sequencing of the parent–child trio. This confirmed both duplications, which were sized as 2.01 Mb (chromosome 16; “DUP16”) and 564 kb (chromosome 17; “DUP17”); both had arisen de novo (Table 1, Additional file 1: Fig. S7). Unexpectedly, examination of the IGV sequence traces at the duplication ends showed that the DUP16 and DUP17 were mutually linked, a conclusion confirmed by breakpoint PCR (Additional file 1: Fig. S8). Analysis of SNV/indel data from the exome and genome sequences established that both DUP16 and DUP17 were of maternal origin, and that each duplicate pair showed an identical pattern of SNVs on the two copies (Additional file 1: Fig. S9). Notably, the DUP17 is positioned near two regions of relevance to the patient’s phenotype: the *ABCA* gene cluster, which has been associated with hypertrichosis [42, 43], and the *KCNJ*-*SOX9* locus, which has been implicated in craniofacial development [44].

**Fig. 3:**
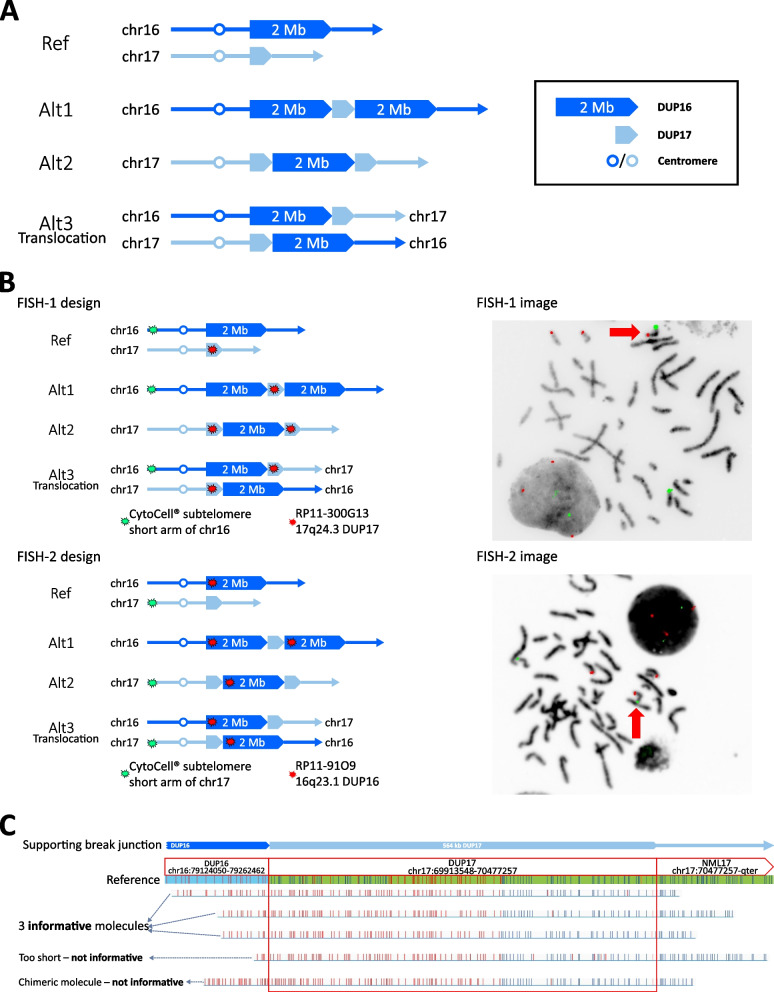
Combined use of FISH and OGM to distinguish three alternative configurations for the Case 2 cxSV. **A** Diagram illustrating three different configurations Alt 1, Alt 2, and Alt 3 (the latter involving a reciprocal translocation between chromosomes 16 and 17), all of which are compatible with the sequence and copy number data shown in Figures S7 and S8. Inset shows terminology for the duplicated segments and relative positions of centromeres. **B** Two-colour FISH analysis by clinical laboratory. The initial design (FISH-1, upper panel; green signal = 16p subtelomeric probe, red signal = probe specific to the 17q duplicated segment) showed an additional DUP17 signal on chromosome 16 (red arrow), interpreted as Alt1 (but also compatible with Alt3). In a later reciprocal design (FISH-2, lower panel; green signal = 17p subtelomeric probe, red signal = probe specific to the 16q duplicated segment), an additional DUP16 signal is present on chromosome 17 (red arrow). The combination of results supports Alt3. **C** OGM analysis. The panel shows the manual realignment of five molecules spanning the 564 kb DUP17, extracted from the Bionano RVA pipeline (see Additional file 1: Fig. S10 for original identification of reads). On review, only the three topmost molecules were considered informative (DUP16-DUP17-NML17 labelling pattern). One molecule, having only four labels in the DUP16 region, was too short to be reliable, while the final molecule has a high labelling density, suggesting it is chimeric (two molecules stuck together and read as one by the machine)

Analogous to the situation described for Case 1, although the work up to this point provided a complete sequence characterisation of the duplication events, it did not resolve the topology of the duplications, for which there were again three solutions (Fig. 3A). Of note, Alt3 would involve a reciprocal translocation between 16q and 17q; as the predicted lengths (13.1 Mb and 13.3 Mb, respectively) and cytobanding patterns of the translocated 16qter and 17qter subtelomeric regions are very similar, Alt3 would not necessarily be detected by standard G-banded karyotyping. Further clinical laboratory investigation was organised using 2-colour FISH, by combining a probe located in the 16p subtelomeric region with a probe specific to the 17q duplicated segment (FISH-1 design shown in upper panel of Fig. 3B). This showed that the 17q probe localised to both chromosomes 17, with a third signal on the long arm of one chromosome 16. This result was interpreted as supporting the Alt1 structure (see Fig. 3A), although it could also be compatible with Alt3.

In parallel with the FISH studies, we undertook Bionano OGM, aiming to find molecules spanning the smaller 564 kb DUP17. Three separate runs were undertaken in an effort to obtain high quality data (coverage: 426.7x, map rate: 92.1%, N50 ≥ 150 kb: 353 kb; Additional file 1: Table S4) and maximise the yield of longer molecules. Using the RVA pipeline to extract all abnormal (non-reference) molecules (Additional file 1: Fig. S10), we identified five molecules spanning DUP17; on closer scrutiny, three of these were considered to yield reliable labelling patterns (Fig. 3C and legend). All three molecules exhibited a DUP16-DUP17-NML17 pattern, compatible with either Alt2 or Alt3 (Fig. 3A), but not with Alt1 as had been clinically reported based on the original FISH-1 experiment. However, no molecules were found to span the much larger DUP16 (2.01 Mb) region, meaning that Bionano OGM data alone could not differentiate between Alt2 and Alt3.

Prompted by the OGM findings, the reciprocal FISH experiment was undertaken by the clinical laboratory (FISH-2, lower panel of Fig. 3B). The combination of FISH and OGM results is only compatible with the Alt3 interpretation (in which the duplications are associated with a reciprocal translocation). The Alt3 (translocation) structure was further confirmed using FISH studies with reciprocal subtelomeric 16qter and 17qter probes (Additional file 1: Fig. S11).

Having established Alt3 as the correct topology, we considered whether the rearrangement could be explanatory of the patient’s clinical features. Interestingly an established locus, *Hypertrichosis, congenital generalised, with or without gingival hyperplasia* (HTC3; OMIM #135400) is localised to 17q24.2-q24.3, coinciding with the DUP17 region. The most distinctive element of the patient’s phenotype is gingival hyperplasia, in addition to which he has localised hypertrichosis with hairy external auditory meati (Additional file 1), providing a good fit to the key features of the HTC phenotype. The DUP17 contains two genes, *KCNJ16* and *KCNJ2*, both of which encode potassium channels, and deepC analysis of the effect of Alt3 on local TAD structure predicts that the regulatory milieu of both genes would be expanded as a consequence of the translocation (Additional file 1: Fig. S12). Although HTC3 has previously been attributed to a biallelic variant in the nearby gene *ABCA5* [42], multiple heterozygous chromosomal rearrangements of this region were previously reported that are inconsistent with this interpretation. Instead, evidence from Case 2 points to misregulation of *KCNJ2* and possibly, *KCNJ16* as the likely underlying pathogenic mechanism and a TAD-based analysis of previously reported chromosome rearrangements is consistent with this conclusion (see Discussion). In summary, we conclude that the Alt3 chromosome abnormality in Case 2 is causative of the patient’s phenotype (see also Additional file 1: Table S5), and provides new insights into the pathogenesis of HTC3.

### Case 3: multiple duplications/triplication on chromosome 20 ranging in size from 31 to 627 kb

Case 3 comprises the female proband, her parents, and maternal grandmother, who were investigated as part of this study. The proband presented with a severe pansynostosis evident at birth and had mild developmental delay; other relevant family members were phenotypically normal (see Additional file 1, Case Descriptions and Analysis, Case 3). Routine genetic investigations did not reveal a cause for the child’s phenotype; aCGH revealed an apparently simple duplication located on 20p12.3p12.2, but this was considered likely to be coincidental to the phenotype as it was inherited from the clinically normal mother. Research-based trio exome sequencing did not reveal any potentially causative variants in the proband.

The family was referred to the 100kGP for genome sequencing of the parent–child trio [25, 27]. Although this failed to identify a separate genetic cause for the pathology, systematic interrogation of the Manta and Canvas datasets re-identified the chromosome 20 CNV (Additional file 1: Table S7) [21] and suggested that it was more complex than had been apparent from the original aCGH. This was further highlighted by plotting the genome coverage data, which revealed two regions of CNV gain; a distal 627 kb region including part of *PLCB4*, and a more proximal 543 kb region that had not been detected by aCGH, which however appeared complex with subregions of both normal, and further increased copy number (Additional file 1: Fig. S13). Integrating both the coverage and the linked reads, four break junctions were identified, splitting the two copy number gains into five segments, four of which were duplicated (sizes 627 kb, 213 kb, 205 kb and 31 kb) and one triplicated (62 kb) (Additional file 1: Fig. S14). Four breakpoints were predicted, and all were confirmed by breakpoint PCR and dideoxy-sequencing (Table 1, Additional file 1: Fig. S15). SNV/indel analysis demonstrated both heterologous, and likely homologous origins for different elements of the duplicated or triplicated sequences (Table 1, Additional file 1: Fig. S16). Analysis of blood-derived DNA from the proband’s phenotypically normal maternal grandmother by breakpoint PCR revealed the same four abnormal breaks (not illustrated), indicating that the cxSV had been inherited through at least three generations, excluding the possibility that the mother was mosaic for the rearrangement.

**Fig. 4:**
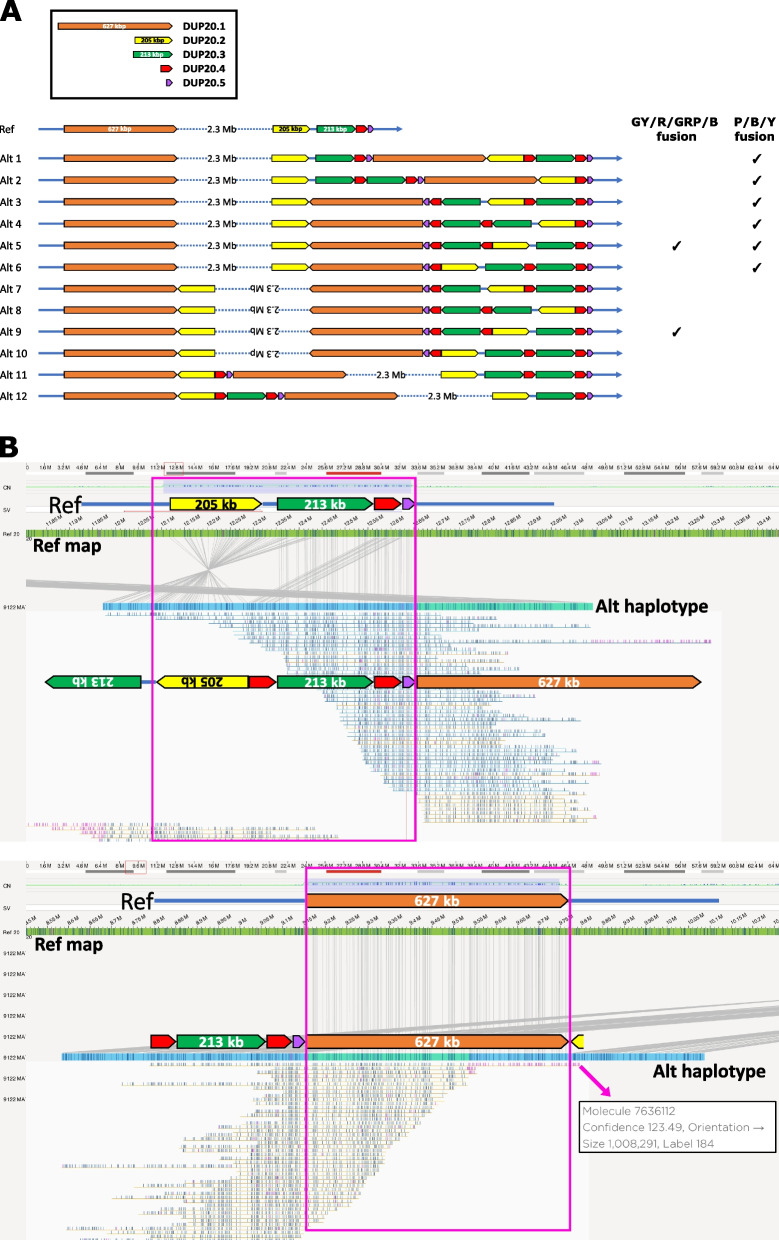
Use of OGM to resolve alternative configurations for the chromosome 20 abnormality present in Case 3. **A** Shows the 12 alternative configurations, Alt1-Alt12, compared to the reference structure (Ref) shown at top. In Alt7-Alt10, the middle 2.3 Mb copy-neutral region lies in inverse orientation. The key to the right shows the structures that are consistent with observations from OGM (G-green; Y-yellow; R-red; P-purple; B-brown;/indicates breakpoint). **B** Bionano OGM data from the unaffected mother of Case 3. The upper panel shows analysis of the more centromeric duplication region (normally -Y-GRP-) and the lower panel the more telomeric region (normally -B-). Red boxes indicate the regions that must be spanned by intact molecules for the data to be informative. In both panels, note a single exceptionally long (1.008 Mb) intact molecule (red callout arrow), which supports the P/B/Y configuration (lower panel)

As with Cases 1 and 2, although this analysis provided a complete molecular description of the breakpoints, it did not resolve the topology. In this case, owing to the larger number of breakpoints, a total of 12 topologies (Alt1 – Alt12) are equally compatible with the data (as the rearrangement has been inherited, the possibility that both chromosome 20 homologues are involved can be excluded) (Fig. 4A). Interestingly, in four topologies, Alt7-Alt10, the central 2.3 Mb copy-neutral region (containing *JAG1*, pathogenic variants of which are rarely associated with craniosynostosis) [45, 46], lies in inverted orientation. We asked whether OGM could help resolve these possibilities.

Interrogating the centromeric, more complex duplicated region first, although this extends over > 600 kb, OGM was readily able to resolve the correct structure (GY/R/GRP/B, see Fig. 4 and legend). This is because the structure is arranged over several individual blocks, none larger than 213 kb, enabling OGM to build up a haplotype using multiple reads spanning each individual segment (Fig. 4B, upper panel). This structure, which is only compatible with Alt5 and Alt9, was also demonstrated in the proband and the proband’s grandmother (not illustrated), indicating stable inheritance of this 3-break rearrangement over 2 transmissions.

In both Alt5 and Alt9, the large brown duplicated sequence (627 kb) is in inverted orientation compared to the native copy, with the intervening normal copy sequence also inverted (together with the yellow duplicated block) in Alt9, but not in Alt5. This could have material impact on regulatory influences on the intervening normal copy sequence, as suggested by deepC analysis (Additional file 1: Fig. S17). To distinguish Alt5 and Alt9 requires OGM reads to span each copy of the 627 kb brown sequence and identify the sequences on either side. In the case of the maternal analysis (Fig. 4B, lower panel, callout arrow) a single, unusually long (1.01 Mb), intact read demonstrated a 627 kb (brown) sequence flanked by breaks to either side (P/B/Y), which in combination with the previous results is diagnostic of Alt5 (Fig. 4A). However further spanning reads to support this interpretation were absent, and in the proband and maternal grandmother no reads were identified that spanned the 627 kb sequence. We conclude that FISH would be required to confirm the Alt5 orientation in the mother and check that the same orientation exists in the proband and maternal grandmother, however this work was beyond the scope of the current study. We also considered the possibility that the proband might harbour a second deleterious allele originating from the paternal chromosome 20 (recessive disease mechanism), but found no evidence to support this possibility. We conclude that, despite disentangling many aspects of this complex chromosome 20p12 rearrangement, its disease classification remains as VUS (Additional file 1: Table S5).

## Discussion

Linked interspersed duplications, and other related complex rearrangements such as triplications, pose a significant technical challenge to determine their correct molecular structure. This phasing problem has previously been highlighted by several authors [7, 12, 13]. The technical difficulty of the challenge, and the most appropriate technology to address the specific problem, will depend on five inherent properties of the rearrangement(s): the number of different elements with increased copy number, the extent to which these elements are themselves linked (rather than being inserted into unique locations in the genome), the size of each of the individual elements, the degree of sequence redundancy of the element, and the molecular process by which the rearrangement has arisen (this determines whether the copied elements comprise identical sequences, or can be differentiated by means of characteristic sequence variants). The three cases represent model problems, which illustrate how the particular combination of the above properties might play out in individual instances. Table 1 summarises key properties of the rearrangements, all of which may be classified as having arisen through the process of chromoanasynthesis [1, 47]. This involves a replicative mechanism of DNA repair (microhomology-mediated break-induced replication; MMBIR), in which template switches occur in the germline mediated by microhomology with a non-homologous region present either (i) at a distance on the same chromosome, (ii) on the autosomal homologue, or (iii) on a non-homologous chromosome [6, 48]. The consequences of this can include the copying of identical sequences (homologous duplication), copying of allelic sequences that exhibit natural variation (heterologous duplication), or translocation, respectively, examples of all of which are documented in the three cases presented.

A specific issue that we address is to ask what specific role OGM (here using Bionano technology) may have in tackling these challenges. Because many interspersed duplications arise through replicative mechanisms that commonly result in identical copies of the duplicated segments (Table 1), the two (or more) copies can then only be distinguished by methods that span the entire duplicated segment in single molecules, to identify the flanking sequences (Fig. 1). Which technology is most appropriate will depend on the length of single molecules that need to be analysed; OGM has the potential to bridge the gap between long-range sequencing methods (for example, nanopore- and single molecule real time [SMRT]-based) [49], which realistically might span tens to low hundreds of kilobases [50, 51], and FISH, for which it becomes increasingly time-consuming to resolve loci separated by under 2 Mb, the interphase resolution limit [52]. Of note, unlike nanopore and SMRT-based methods, which achieve base-pair resolution and should reliably document any SNVs/indels that could be used to distinguish non-identical duplicate copies, OGM will struggle to distinguish them—even if they contain polymorphic differences—owing to intrinsically poor resolving power (~ 500 bp) and to the dropout of individual signals on single molecules, making it more difficult to determine whether observed signal variation is attributable to genuine polymorphic difference or simply to signal dropout.

Example Case 1 comprised a relatively simple scenario involving two large linked direct duplications (a third much smaller [148 bp] inverted duplication was present, but this did not substantially alter the underlying issues). Nevertheless the relatively large size of the duplicated segments (244 kb and 323 kb) made it challenging to deduce the correct structure. Importantly, when the OGM analysis was run in automated mode, the structure that emerged was incorrect (Additional file 1: Fig. S4). This was because the automated software piled up large numbers of molecules that contained single breakpoints, but did not fully span the duplicated segment. By following this process on either side of the duplicated segment, the two sets of molecules met in the middle and seemingly created a contiguous map; however, it later became apparent that this map forced together molecules that did not lie on the same strand, driving a phase switch. This error became apparent when we obtained higher quality DNA and filtered the data to include only reads completely spanning the shorter (244 kb) duplicated segment; the deduced structure (Alt2) was independently confirmed by FISH. Notably, the 244 kb segment derived from two identical (paternally-originating) copies, making derivation of phase using internal markers impossible (Table 1). By contrast, the 323 kb segments are derived from the two different paternal copies, which might have afforded opportunities for phasing using nanopore or SMRT sequencing, but would be very challenging for OGM.

Example Case 2 also comprised two de novo duplications, however this presented a distinctly different problem because the duplications lay on different chromosomes. Assumed from aCGH to be independent events, genome sequencing showed that the duplications were in fact physically linked. Initially it seemed most parsimonious to assume that a single chromosome was involved, with one of the duplicated sequences inserted into the other chromosome and associated with a simultaneous duplication of material on that chromosome (ie possibilities Alt1 or Alt2 in Fig. 3A). Indeed, a clinical FISH test, showing the insertion of chromosome 17 material into chromosome 16 (Fig. 3B), was thought to be compatible with Alt1 and reported as such.

This case represented a much greater challenge for OGM, because of the larger sizes of the duplicated segments (564 kb and 2.01 Mb). Spanning the 2.01 Mb segment was not feasible, but we were able to generate a small number of molecules that completely spanned the 564 kb chromosome 17 segment, that were not sourced from the normal gene copy because the banding pattern changed on one side of the duplication, but not the other (Fig. 3C). This pattern was not compatible with the clinical lab report; rather, in combination with that report, it suggested Alt3 (translocation), which was subsequently confirmed by FISH. Although the OGM was extremely helpful here, it was nevertheless suboptimal, because of the paucity of molecules obtained that fully spanned the 564 kb region. In particular, we failed to identify any molecules characteristic of the duplicated region on the rearranged chromosome 17, present in both the Alt 2 and Alt3 product (NML17-DUP17-DUP16 labelling pattern in Fig. 3A). Moreover, given the variation in labelling between individual molecules, and the existence of molecules stuck together yielding chimeric patterns, our interpretation was qualitative rather than rigorously quantitative. A broader question, important for clinical application, would be to determine when a particular set of OGM labelling data represent a specific truth, beyond reasonable doubt. In this case, more extensive analysis with high molecular weight DNA, to generate additional molecules fully spanning the DUP17, would have been necessary for OGM to reach that threshold. Even then, OGM could not distinguish Alt2 and Alt3, because that would require spanning the larger (2.01 Mb) DUP16; FISH was much better suited to reaching a definitive solution (Figs. 1, 3B and Additional file 1: Fig. S11). A final point to note is that in this case, the two chromosome 16 and chromosome 17 copies appeared identical (Additional file 1: Fig. S9), so long-read sequencing would have been unable to distinguish the duplicates by phasing SNVs or indels. Analogous translocation-associated duplications were previously described in the context of several breast cancer cell lines [53], but the reciprocal pattern observed in Case 2 appears to be very rare in the context of constitutional disease.

The final Case 3, comprising a combination of four duplications and one triplication originating from adjoining regions of chromosome 20 and inherited through three generations, posed a different set of challenges. Whilst the initial assessment of structural possibilities identified a much more complex picture with 12 alternative arrangements (Fig. 4A), OGM showed its utility when confronted by a problem suited to its strengths, by excluding all but two of the structures without any difficulty. This was because four of the five duplicated/triplicated segments ranged in size from 31 to 213 kb, and it was straightforward for OGM to generate informative molecules spanning each of these segments individually and thus identify the correct sequence of elements. By contrast, particularly since two of the duplications were nevertheless substantial in size – 205 kb and 213 kb – and the 213 kb duplications appeared identical in sequence (Additional file 1: Fig. S16), this would be a very challenging problem for either nanopore or SMRT sequencing to solve. Resolving the two final structures required the ability of OGM to span the largest duplicated segment (627 kb), and, at least in our hands, this did not prove routinely feasible. Therefore, this case remains incompletely resolved. Given that the cxSV was present in at least three generations, with a normal phenotype in mother and grandmother, it has been considered that this rearrangement has low clinical priority for further workup. However, we note the theoretical possibility that the inverted orientation of the 627 kb duplicated elements in both Alt5 and Alt9 could predispose to a further inversion arising between generations, giving rise to flipping between these alternative structures, in which the involved genes reside in different regulatory milieu (Additional file 1: Fig. S17). Further FISH studies would be required to investigate this possibility.

A critical issue that we encountered across all three cases was that the Bionano default/automated analysis pipeline for constitutional SVs, i.e. de novo assembly analysis, was not suited to characterise these large cxSVs. This limitation arose from two key aspects of how the Bionano OGM analysis is currently set up. Firstly, the de novo assembly pipeline recommends > 80X coverage and N50 > 230 kb (see Optical genome mapping using the Bionano platform in Material and Methods), which is inadequate for capturing enough spanning informative molecules for the example cases – namely > 500 kb for Case 2 and > 600 kb for Case 3. To improve the probability of capturing such molecules, one may increase the N50 by reducing DNA fragmentation during DNA extraction. However, this approach also significantly increases the risk of molecules adhering to one another, clogging the chip and causing it to fail. As an alternative, we escalated the throughput and coverage to the limit of the chip (~ 400x), keeping the N50 at an optimal size for the chip performance while also increasing the likelihood of capturing long informative molecules. Secondly, the default constitutional analysis pipeline (de novo assembly pipeline) performs down-sampling at random for high coverage data (> 250x: [“Bionano Solve Theory of Operation: Structural Variant Calling”, Document Number 30110, Revision K]), which undermines the purpose of increasing coverage. In contrast, the RVA pipeline—originally designed to identify rare variants in cancer applications—extracts all abnormal (non-reference) molecules, providing a much more thorough examination of high-coverage data, potentially allowing all informative molecules to be captured if present in the library.

To illustrate the general properties of the OGM analyses, we quantified the size distribution (Additional file 1: Fig. S18A) and number of molecules (Additional file 1: Fig. S18B) for each of the three cases. Above 200 kb the number of molecules declined by 35–55% for each 100 kb bin, with longer molecules better enriched in Case 3 > Case 2 > Case 1. To determine how robustly we could detect each of the 10 abnormal duplicated segments in our case series, we quantified the absolute number of molecules diagnostic for each of the 14 types of rearrangement present (Table 2). As expected the 148 bp DUP13.2 was not detectable using Bionano OGM, which has a minimum resolution of ~ 500 bp. The DUPs ranging in size from 31 to 244 kb were all readily characterised using multiple spanning reads. Characterisation of the 323 kb and 563 kb DUPs was also feasible, but even after maximising DNA quality and data acquisition, only 0–3 reads were obtained for each rearrangement type. As previously described, only a single read was obtained across the 626 kb DUP20.1 combining the three individuals analysed (Fig. 4B). In our hands, therefore, the upper size limit for Bionano OMG to characterise interspersed duplications was ~ 550 kb.

Whilst the principal focus of this report has been to explore the technical aspects of structure disambiguation, the ultimate aim of our work was to provide robust molecular diagnoses for patients who had a previously undetermined cause of their clinical presentation with syndromic craniosynostosis. We were interested in documenting both the gene content of the rearrangements that we identified, and also how the different order and/or orientation of these rearrangements might impact on the local TAD structure. This “TAD-oriented” approach has proved powerful in determining the pathogenic mechanism of a select group of large-scale rearrangements, including some in patients with craniosynostosis [23, 54, 55]. To undertake this analysis we used deepC, a transfer-learning-based deep neural network that accurately predicts genome folding from megabase-scale DNA sequence [33], to explore how alternative chromosome structures would likely impact the regulation of neighboring genes. With the caveat that the IMR-90 fetal lung fibroblast line only provides a proxy for the many different cell types resident in the cranial sutures, this approach was useful for exploring disease hypotheses in all three families, and in two (Cases 1 and 2), functional or genetic analysis further supports the instructiveness of a TAD-based approach for elucidating disease mechanism.

In Case 1 we predicted, based on the Alt2 structure, that *FGF9* could come under the influence of the inserted 244 kb and 323 kb DUP sequences in the 13q12.11 rearrangement within an expanded TAD, including sequence annotated as having craniofacial superenhancer activity (Additional file 1: Fig. S6) [41]. This prediction was recently supported by functional studies demonstrating increased *FGF9* expression and more open chromatin in neural crest cells derived from the affected individual (Korona D, Hashimoto AS, Pei Y, Calpena E, Sloane-Stanley J, Riva SG, Schwessinger R, Forzano F, Chintawar S, Duggal G, Wall SA, Hughes JR, Twigg SRF, Wilkie AOM: Evaluating the pathogenic significance of unique chromosomal variants in craniosynostosis using patient-derived induced pluripotent stem cells and mouse modelling, submitted).

**Fig. 5:**
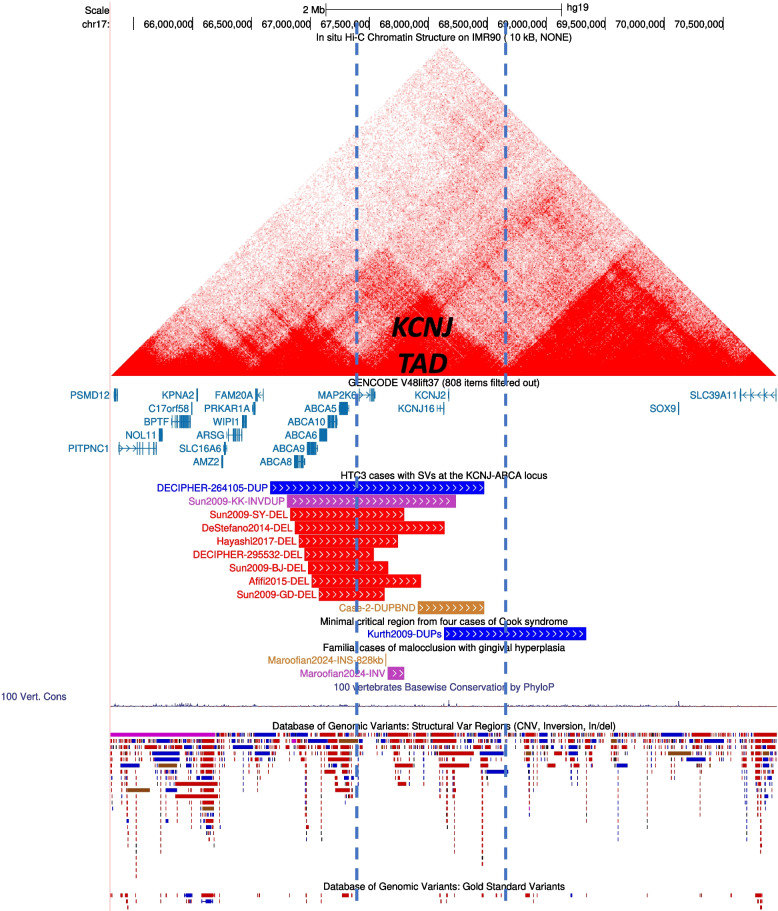
Rearrangements of the 17q24.3 region associated with abnormal phenotypes. Shown below the corresponding genes in the UCSC Browser [61], the CNVs (red- deletions; blue- duplications; purple, inverted duplication), which were extracted from the publications by Sun et al. [43], DeStefano et al. [42], Afifi et al. [62], Hayashi et al. [63], Maroofian et al. [57] and DECIPHER, are associated with hypertrichosis and/or gingival hyperplasia. The duplications published by Kurth et al. [64] (minimal critical region is depicted) are associated with Cooks syndrome. The DUP-translocation identified in Case 2 is shown as the brown segment. Above, the main *KCNJ* TAD (IMR-90 data from Rao et al. [34]) is flanked by blue dashed lines. Note that the 5’ (centromeric) boundary deleted in multiple HTC/GH patients is classified as ultra-conserved, being identifiable across multiple primate and rodent species [58]. Below, the DGV track demonstrates that rearrangements similar to the featured CNVs do not occur as common variants. Figure in hg19. UCSC session: https://genome.ucsc.edu/s/429035671/Pei_et_al_2025_fig5

Whereas the chromosome rearrangement and clinical presentation of Case 1 currently appears unique in the literature, a TAD-based analysis of Case 2, in which the proband is affected by localised hypertrichosis (HTC) and gingival hyperplasia (GH) and harbours a duplication of 17q24.3, is more broadly instructive for disease mechanism at the *HTC3* locus. *HTC3* was previously localized to 17q24.3 (OMIM 135400), where GH is an additional feature of the phenotype. Although the underlying cause has been attributed to *ABCA5* as a recessive disease gene, only one case, concerning a homozygous splice site variant has been reported [42]. The failure to replicate this observation in any additional families over the past decade, and the tabulation of four different homozygous loss-of-function *ABCA5* variants (each in a single individual) in gnomAD v4.0.0 [56], raises the possibility that this could have been a coincidental finding. More compellingly, multiple heterozygous deletions and other rearrangements of the 17q24.3 region (both including and excluding *ABCA5*) associated with HTC ± GH have been published, confirming that a disease locus lies within the broader region (Fig. 5) [57]. In Case 2, the start of the DUP17 is separated by ~ 586 kb from *ABCA5* but the duplication contains two genes, *KCNJ16* and *KCNJ2*, both of which encode potassium channels. Given that the previously described 17q24.3 rearrangements are all predicted to disrupt an ultra-conserved TAD boundary on the centromeric side of *KCNJ16* and *KCNJ2* (Fig. 5) [58], the most parsimonious explanation of the data is that the described rearrangements drive altered expression of one or both of *KCNJ16* or *KCNJ2* (*KCNJ16* was included in the deletion reported by DeStefano et al. [42], suggesting that *KCNJ2* is more likely to be the key driver gene). Consistent with this hypothesis, pathogenic variants in other members of the potassium channel superfamily (*KCNH1* and *KCNN3*) were previously reported in the clinically related condition Zimmerman-Laband syndrome (ZLS), a phenotype combining gingival fibromatosis and terminal deficiency of the digits (loci ZLS1 and ZLS3, respectively). A further overlap is provided by the phenotype of Cooks syndrome, which is characterised by terminal deficiency of the digits reminiscent of that in ZLS; this was previously attributed by experimental modelling in mice to misregulation of *Kcnj2* (the murine orthologue of *KCNJ2*), mediated by large duplications located on the telomeric side of *KCNJ16*/*KCNJ2* [59]. In summary, the combined OGM/FISH analysis of Case 2 has established a causal link between the chromosomal rearrangement and the HTC phenotype, provided key evidence towards a novel hypothesis of HTC3 causation [57], and shown that counselling of offspring risk for the affected individual will be not for a simple dominantly inherited condition, but a reciprocal (but unbalanced) translocation.

Although this study has rigorously tested the strengths and weaknesses of Bionano OGM in three particularly challenging cases, it has clear limitations. The underlying sample size comprising 14 different abnormal duplication topologies (Table 2) was relatively small. We did not perform long-read sequencing (either SMRT- or nanopore-based) in parallel, so cannot directly compare the efficacy of those technologies in solving these difficult cases. However we have summarised (Table 1) the respective sizes of each duplication, together with whether each arose through a heterologous versus homologous mechanism, from which the applicability of other technologies can be surmised based on their own size limits and ability to detect allelic variation. Neither did we systematically compare the results with the routine use of FISH, although we did verify the structures in Cases 1 and 2 using this method. The FISH approach was time-intensive in Case 1 (see description in Additional file 1 and Additional file 1: Fig. S5) and we anticipate that it would have been even more so (given the complex structure of the chromosome 20 rearrangement) in Case 3, whereas in Case 2 FISH provided the simplest route to a definitive solution (Figs. 1, 3A, B and Additional file 1: Fig. S11).

## Conclusions

This work illustrates how OGM (undertaken here using the Bionano instrument) can be very useful in solving ambiguous structures involving elements between tens of kilobases and ~ 550 kb in size. Below this size range, long-read sequencing [50] provides clear advantages because of base-pair accuracy. Expanding the upper size range of OGM will depend on increasing the yield of very long (500 kb → 1 Mb) labelled molecules. Our identification of a single molecule of 1.008 Mb in Case 3 (Fig. 4B) shows that this is achievable, and improvements in DNA preparation and analysis chips may make analyses at this scale feasible in the near future. Above the 500 kb-1 Mb size range, application of FISH techniques is likely to remain preferable. Whilst sequence capture methods [60] can also operate within these size ranges, interpretation of the data is likely to be more challenging in achieving unambiguous ordering of sequence pieces.

## Supplementary Information

Additional file 1: Comprises 7 supplementary Tables, 18 supplementary Figures, clinical reports for Cases 1-3, and supplementary references. This file provides a detailed molecular characterisation of each rearrangement, together with information on FISH probes, PCR primer pairs, Bionano quality metrics and the pathogenicity classification of each rearrangement

## Data Availability

All datasets generated during this study are either included in this published article and associated Additional file 1, or have been submitted to the ENA under accession number PRJEB73282, [https://www.ebi.ac.uk/ena/browser/view/PRJEB73282 and https:/www.ebi.ac.uk/ena/browser/view/PRJEB73282?show=analyses (Bionano read data)] [30]. Parent–child trio exome and/or genome sequence data for Cases 2 and 3 have been deposited in the ENA, accession PRJEB73282 [30]. De-identified genome sequence data for Cases 1 and 3 are available within the Genomics England Research Environment, subject to a collaborative agreement that adheres to patient led governance. All interested readers will be able to access the data in the same manner that the authors accessed the data. For more information about accessing the data, interested readers may contact research-network@genomicsengland.co.uk or access the relevant information on the Genomics England website: https://www.genomicsengland.co.uk/research.

## Abbreviations

100kGP: 100,000 Genomes Project
aCGH: Array comparative genomic hybridisation
ACMG: American College of Medical Genetics
CNV: Copy number variant
cxSV: Complex structural variant
DUP: Duplicated segment
FISH: Fluorescence in situ hybridisation
GH: Gingival hyperplasia
IGV: Integrative Genomics Viewer
Indel: Insertion/deletion variant
INV: Inverted segment
iPSC: Induced pluripotent stem cell
HTC: Hypertrichosis and gingival hyperplasia
MMBIR: Microhomology-mediated break-induced replication
NML: Normal orientation and copy number
N50: Length of the shortest molecule such that all molecules of that length or longer collectively cover 50% of the total assembled sequence
OGM: Optical genome mapping
RE: Research Environment
REC: Research Ethics Committee
SMRT: Single molecule real-time
SNV: Single nucleotide variant
TAD: Topologically-associating domain
VUS: Variant of uncertain significance
