# Supplementary material for: Exploring the size limits of Bionano optical genome mapping to resolve alternative structures of linked interspersed chromosomal duplications
Source: Genome Med. 2025 Nov 13;17:141. doi: 10.1186/s13073-025-01571-0 (PMC12616954; doi:10.1186/s13073-025-01571-0)
Supplement: Supplementary file 1 — Additional file 1: Comprises 7 supplementary Tables, 18 supplementary Figures, clinical reports for Cases 1-3, and supplementary references. This file provides a detailed molecular characterisation of each rearrangement, together with information on FISH probes, PCR primer pairs, Bionano quality metrics and the pathogenicity classification of each rearrangement [file 13073_2025_1571_MOESM1_ESM.pdf]

Exploring the size limits of Bionano optical genome mapping to resolve alternative structures of linked interspersed chromosomal duplications, by Yang Pei *et al*.

Additional file 1: Supporting Information

Contents **Page**

Table S1: Probes and fluorophores used for FISH analyses 2

Table S2: hg19/GRCh38 conversions for each duplication end 2

Table S3: Primer pairs used for breakpoint PCR 3

Table S4: Quality metrics for each Bionano sample analysis 4

Table S5: Pathogenicity of variants in Cases 1-3 assessed using ACMG criteria 5

Figs S1-S6, Table S6: Case 1, Clinical phenotype and additional molecular investigations 6

Figs S7-S12: Case 2, Clinical phenotype and additional molecular investigations 15

Figs S13-S17, Table S7: Case 3, Clinical phenotype and additional molecular investigations 24

Fig. S18: Size distribution and absolute number of molecules analysed in Cases 1-3 34

Supplementary References 35

Table S1: Probes and fluorophores used for FISH analyses.

BACs	Genomic Coordinates hg19	Genomic Coordinates hg38	Label
RP11-441F14	chr13:22855636-23039514	chr13:22281497-22465375	Cy3
RP11-1095B20	chr13:23323910-23587290	chr13:22749771-23013151	DIG
RP11-1013L17	chr13:24223379-24423559	chr13:23649240-23849420	AF647
RP11-300G13	chr17:68027658-68214259	chr17:70031517-70218118	R-ROX
RP11-91O9	chr16:78053567-78205164	chr16:78019670-78171267	R-ROX

Table S2: hg19/GRCh37 to hg38/GRCh38 conversions for each duplication end.

Case	hg19/GRCh37	hg38/GRCh38
1	chr13:22796750-23119988	chr13:22222612-22545849
	chr13:23886689-23886837	chr13:23312551-23312698
	chr13:24183826-24428230	chr13:23609688-23854091
2	chr16:77292432-79301239	chr16:77258535-79267342
	chr17:67909691-68473397	chr17:69913550-70477256
3	chr20:9134127-9761066	chr20:9153480-9780418
	chr20:12095748-12300361	chr20:12115100-12319713
	chr20:12331358-12544702	chr20:12350710-12564055
	chr20:12544703-12607144	chr20:12564056-12626497
	chr20:12607145-12638362	chr20:12626498-12657715

Table S3: Primer pairs used for breakpoint PCR.

Case	Primer	F/R	Oligo 5'-3'	Product length (bp)
1	C1-DL-F	F	CATAGAAAAATCTTGGTAATTGGC	403
	C1-DL-R	R	TGACAGTAGCTGCTTAATTCAGG	
	C1-LTD-F	F	GTATTGTCTACTTTAGATATCAGC	415
	C1-LTD-R	R	TCGTTAATATCTTACAACCATCC	
2	C2-LD-F	F	AATCACTCTATGGAGGGTCCAGC	197
	C2-LD-R	R	CTTCCACTTATGCTTACCCCTGC	
	C2-DL-F	F	CTGTCATTCCCACAGTACATATTGCC	474
	C2-DL-R	R	GGGGACACAACCAAACCATATTAGC	
3	C3-RG-F	F	TCAGCAAGGAAGCCATCAGG	485
	C3-RG-R	R	GAAGTGCTGCTCCTTGAATTGC	
	C3-PB-F	F	GCTCTGCTAATAATGGTTTGGCTC	451
	C3-PB-R	R	CTGAATGAAAACGTACCTGGGC	
	C3-BY-F	F	ACCATATTTAAGCTAGGCTCCC	505
	C3-BY-R	R	TGAGCCTAAAAAAGATCCCCC	
	C3-YR-F	F	CTTTCACCTTCGCTGTGTTGG	691
	C3-YR-R	R	CTCCATTTGCCTACCAATTTCCC	

Table S4: Quality metrics for each Bionano sample analysis.

Case	1	2	3	3Mat[a]	3MatMat[a]
**CRS ID**	**S474**	**347**	**302**	**302**	**302**
**Total DNA (≥20 kb), Gb[b]**	1371.4	1926.2	1855.6	2027.1	2194.6
**Total DNA (≥150 kb), Gb[b]**	867.1	1504	1525.6	1504.4	1535.5
**N50 (≥ 20 kb), kb[c]**	217.1	292.2	290.6	253.2	222.7
**N50 (≥150 kb), kb[c]**	348.9	352.9	333.5	315.7	286.9
**Average label density (≥150 kb), /100 kb[d]**	18.0	15.1	15.6	15.0	15.5
**Map rate (≥150 kb)[e]**	79.5%	92.1%	94.7%	91.9%	92.6%
**Effective coverage (x)[f]**	217.75	426.68	450.36	430.46	442.83
**Molecule integrity number[g]**	0.17	0.09	0.07	0.07	0.07
**Positive label variance (PLV)[h]**	2.7%	3.0%	2.3%	2.1%	2.5%
**Negative label variance (NLV)[i]**	7.5%	8.0%	6.8%	10.8%	6.7%

[a]3Mat and 3MatMat refer respectively to the mother and maternal grandmother of Case 3. [b]Total amount of DNA from molecules that are 20 and 150 kb or longer, respectively; [c]For DNA molecules that are 20/150 kb or longer, respectively, N50 is the length of the shortest molecule such that all molecules of that length or longer collectively cover 50% of the total assembled sequence. [d]Average number of labels per 100 kb for the molecules that are 150 kb or longer; [e]Percentage of molecules that are 150 kb or longer mapped to the reference; [f]Total amount of aligned DNA divided by the size of the reference genome times the map rate; [g]A measure of single molecule quality, where lower values are preferred and should be less than 20; [h]Percentage of labels absent in reference; [i]Percentage of reference labels absent in molecules.

Table S5: Pathogenicity of cxSVs in Cases 1-3 assessed using ACMG criteria, including adapted criteria for non-coding regions.

	CNV-based criteria[a]						Adapted ACMG non-coding variant criteria[a]			
	2A: Overlap with established triplosensitive gene	4B: De novo occurrence, phenotype consistent with genomic region, specific but not unique	4C: De novo occurrence, phenotype consistent with genomic region, not specific	4J: Variant present in individual unaffected by the specific phenotype in proband	Total	Initial interpretation	De novo occurrence	Computational	Functional	Final interpretation
Case 1	-	-	0.15	-	0.15	VUS[b]	PS2	PP3	PM1	Likely pathogenic
Case 2	1[c]	0.3	-	-	1.3	Pathogenic	na[d]	na	na	Pathogenic
Case 3	-	-	-	-0.3	-0.3	VUS	na[d]	na	na	VUS

[a]American College of Medical Genetics (ACMG) criteria for CNVs were applied according to Riggs et al. (2020) [1]; for Case 1, criteria for non-coding regions were applied using the proposals described by Ellingford et al. (2022) [2] to modify criteria originally developed by Richards et al. (2015) [3].
[b]VUS, variant of uncertain significance.
[c]*KCNJ2* has a pTriplosensitivity score of 0.948 according to Collins et al (2022) [4]. ClinGen currently annotates this region as having a TS score of 1 (little evidence of triplosensitivity; https://search.clinicalgenome.org/kb/gene-dosage/region/ISCA-46303#report_details_triplosensitivity), however the pathogenic mechanism in Case 2 is likely novel, acting through altered gene expression (see Discussion).
[d]na, not applied in Cases 2 and 3 (no functional analysis was performed).

Supplementary Case Descriptions and Analysis

Case 1 (family ID S474)

Clinical phenotype and additional molecular investigations

The female patient, currently aged 9 years, is the only child from her parents' union. The mother is affected with a progressive form of spinocerebellar ataxia. The family history is otherwise unremarkable. At birth there was marked dolichocephaly with a prominent sagittal ridge and a small anterior fontanelle. There was a skin defect overlying the anterior skull, extending along the forehead and down onto the nasal bridge. The ears were low-set and the auditory channels were hypoplastic. Computed tomography scan detected a fused sagittal suture, for which she underwent cranial surgery at the age of 9 months. She has shown global developmental delay. She started walking at 2.5 years, she has acquired a very limited vocabulary, and she remains in nappies. She suffers from constipation. She underwent multiple otorhinolaryngology interventions to fit bilateral bone-anchored hearing aids due to her meatal atresia. Her general health is otherwise good.

Genetic investigation of the proband included array comparative genomic hybridisation (aCGH) analysis using oligonucleotide arrays with ~60,000 probes across the genome (Agilent design 028469). This was reported as arr 16q12.2q13(56,660,984-56,704,263)x3 pat [hg19], based on the identification of a ~43 kb duplicated segment on chromosome 16, shown by subsequent targeted array analysis to have been paternally inherited and therefore likely coincidental to the patient's phenotype. No other imbalance (excluding established population polymorphisms) was detected. The results were consistent with a female chromosome complement. Review of the microarray result following genome sequencing revealed that the larger duplicated segments on chromosome 13 (244 kb and 323 kb) each contained only three probes, and although increased signal for these probes was evident, this was not called by the clinical array software.

In addition to the chromosome 13 rearrangements described in this work, analysis of the genome sequence from the 100,000 Genomes Project identified a de novo single nucleotide deletion within the *FOXP2* gene, NM_014491.4:c.982del encoding p.(Arg328Glufs*37), predicting

heterozygous loss-of-function. Similar pathogenic variants of this gene have been described in association with speech apraxia [5] and rarely in craniosynostosis [6], raising the possibility that the patient may have two separate explanations of their pathology.

FISH analysis

A total of 334 cells (147 control with 274 allele signals; 187 patient with 371 allele signals) were examined to determine the true configuration of the Case 1 complex structural variant (cxSV). The majority (65%) of the signals were non-informative, predominantly signals from likely replicating cells, but also including overlapped, overexposed, or noisy signals (non-specific hybridisation). From the control sample, 90% informative signals supported the reference pattern, one signal had a pattern resembling Alt2, and 9% informative signals did not support either the Ref or the Alt patterns. From the patient sample, 47% informative signals were consistent with the reference pattern, suggesting the patient is likely heterozygous, and ruling out the Alt3 configuration. Another 31% signals showed an Alt2 or likely Alt2 pattern, and only one signal was most likely the Alt1 interpretation. A total of 21% signals were not consistent with either Ref or the Alts, and these were likely partially non-informative signals (poor probe hybridisation). Fig. S5B shows three example FISH images supporting the Alt2 configuration, with each cell containing one reference signal and one Alt2 signal. Only one informative cell from the patient sample showed a reference pattern on both alleles, excluding a high level of mosaicism of cells with a normal chromosome 13 complement. Combining the results of all genomic analyses, the molecular notation for the chromosome 13 rearrangement was concluded to be:

NC_000013.11:g.22545849_22545850ins[23312552_23312698inv;A;23609688_23854091;22222612_22545849].

Table S6: Abnormal Canvas and Manta calls located on chromosome 13 from genome sequencing data of Case 1.

CHROM	POS[a]	END	SVTYPE	SVLEN	ID	QUAL
**chr13**	22796455	23119182	CNV	.	Canvas:GAIN:13:22796455:23119182	61
**chr13**	22796728	24428206	DUP	1631478	MantaDUP:TANDEM:108316:0:1:0:0:0	671
**chr13**	24183830	24427999	CNV	.	Canvas:GAIN:13:24183830:24427999	61
**chr13**	23886691	24183826	INV	297135	MantaINV:108353:2:3:0:0:0	742

[a]Co-ordinates based on hg19/GRCh37 reference

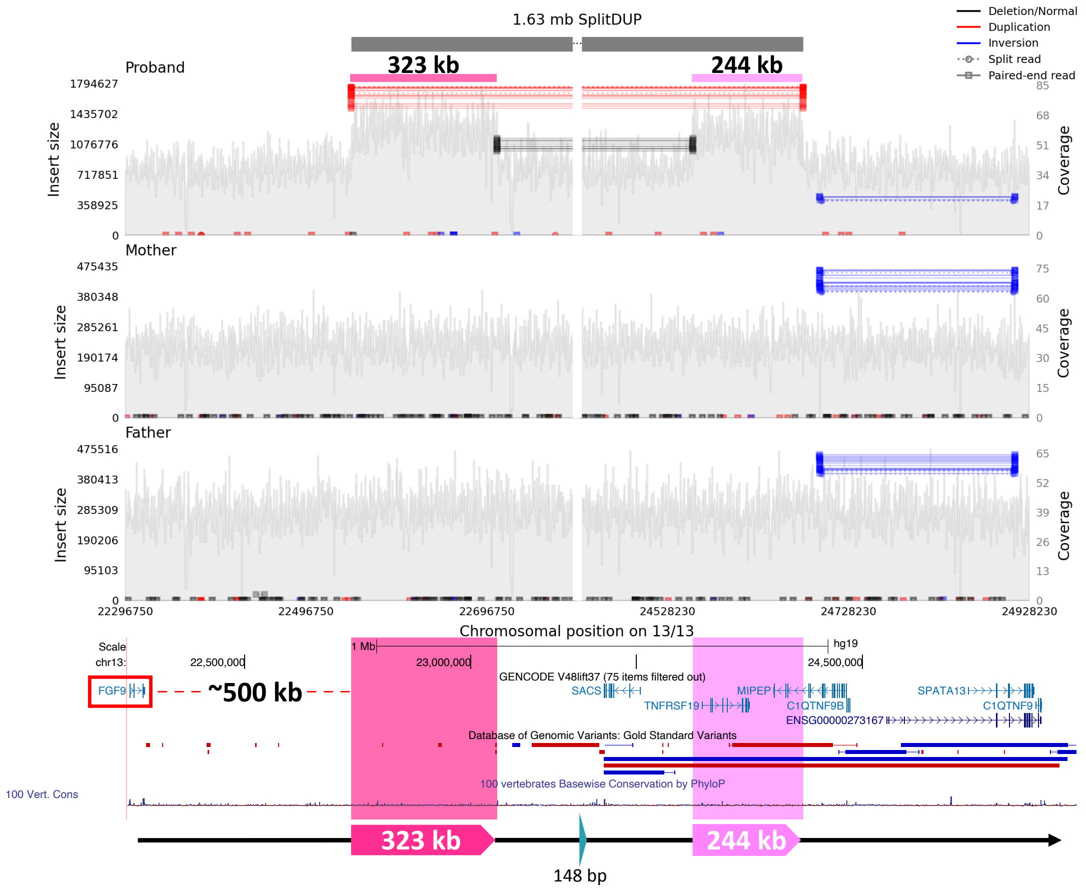

Fig. S1. Interspersed de novo duplications on chromosome 13 in Case 1 revealed by genome sequencing. The upper panels show sequence coverage in (from top) the proband, mother and father, based on Samplot analysis. The coverage depth (right *y*-axis) is plotted against genomic coordinates (*x*-axis), with duplicated regions expected to show elevated coverage levels compared to the normal baseline (~30×). Separately, reads are plotted by insert size (left *y*-axis) against genomic coordinates. Abnormal reads for this split-duplication (those coloured red and black) predict increased insert sizes and appear higher on the *y*-axis, while normal reads aggregate at the baseline, reflecting the typical ~150 bp insert size of Illumina short-read sequencing. Abnormal reads are displayed in “jitter” mode to illustrate the presence of multiple overlapping pairs. The lower panel shows screenshots from the University of California Santa Cruz (UCSC) Genome Browser and relates the positions of the two large duplications (323 kb; dark pink and 244 kb; light pink) to local gene content, 100 vertebrate sequence conservation, and CNVs from the Database of Genomic Variants (DGV; red horizontal lines – deletions; blue lines - duplications). Session for the UCSC track is saved as: https://genome.ucsc.edu/s/429035671/Pei_et_al_2025_figS1_UCSC_track. For clarity, note that the ~1.1 Mb central segment between the two large duplications (including the small 148 bp duplication; teal) is omitted. The numbering shown uses the hg19 reference genome build in which the genome sequence data were originally analysed.

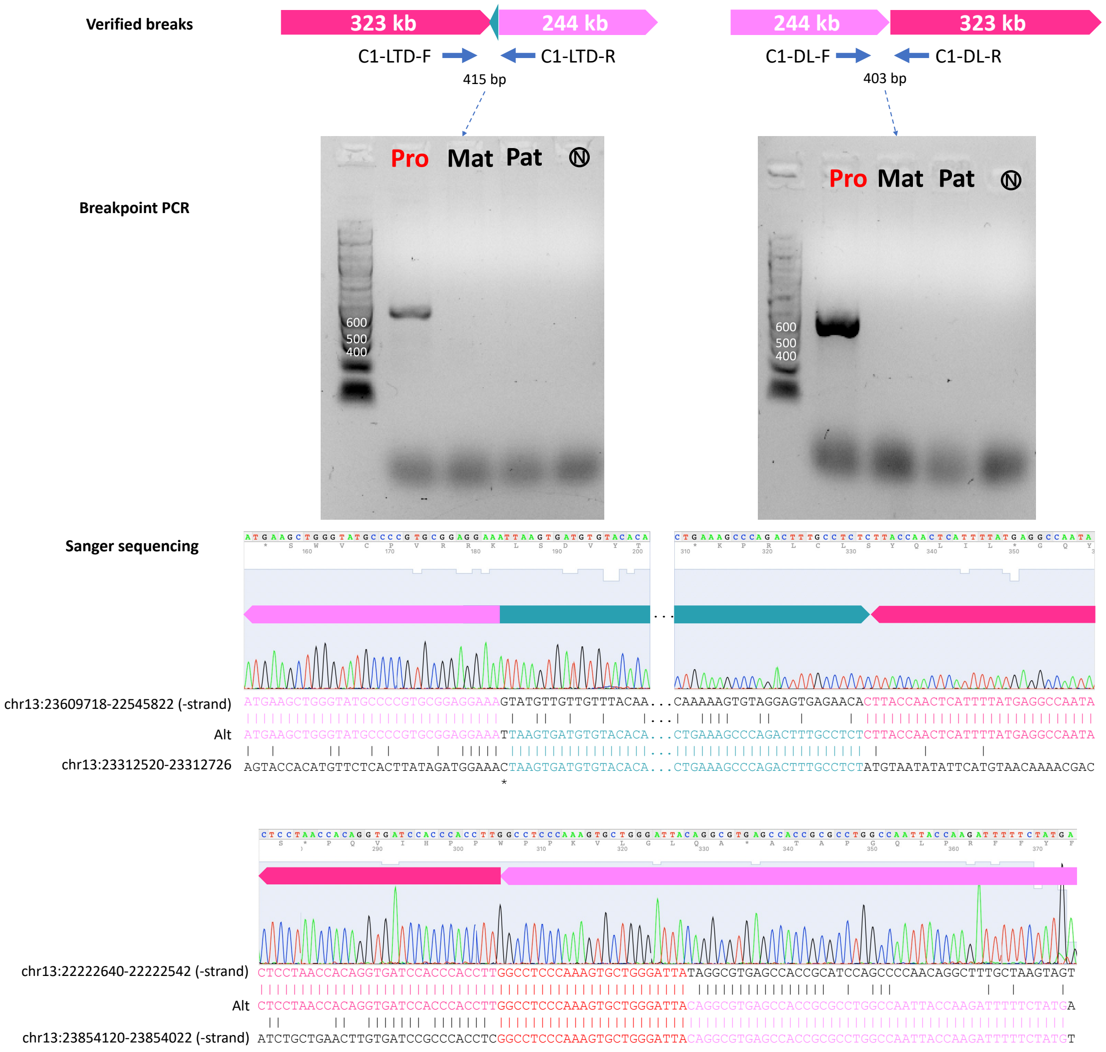

Fig. S2. Breakpoint PCR and dideoxy-sequence based verification of the three break junctions in Case 1. Top panel shows diagrammatically the positions of the breakpoint PCR primers (Table S3) used to amplify unique products in the proband (Pro), but not the parents (Mat, mother; Pat, father; N, no DNA control), demonstrated by agarose gel electrophoresis (central panel), confirming the de novo nature of both events. Bottom panels show the sequences at the breakpoint junctions and indicate extent of homology at the sequence joins. *Indicates the site of a C/T single nucleotide variant (SNV) rs11616235, at which Case 1 inherited the non-reference T allele. Sequence coordinates were mapped to hg38.

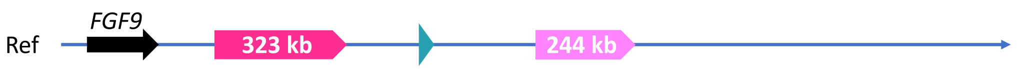

SNP position on chr13 (hg38)		22816548	22823487	22829381	22845691	22862648	22863360	22912251	23003123	23003450	23007750	23008281	23009418	23011943	23017653	23312551	24207224	24207404	24316167	24316821	24321261	24341826	24396908	24443810
Proband		T	A	A	T	C	A	del	G	A	T	G	T	C	G	C	C	C	T	A	T	del	C	TTTTA
		T	A	A	T	C	A	del	G	del	C	A	C	T	C	T	T	T	del	G	C	AG	A	del
		G	G	G	C	T	G	C	T	del	C	A	C	T	C	C	T	T	del	G	C	AG	A	del
Mother		T	A	A	T	C	A	del	G	A	T	G	T	C	G	C	C	C	T	A	T	del	C	TTTTA
		T	A	A	T	C	A	del	G	A	T	G	T	C	G	T	C	C	T	A	T	del	C	TTTTA
Father		T	A	A	T	C	A	del	G	del	C	A	C	T	C	T	T	T	del	G	C	AG	A	del
		G	G	G	C	T	G	C	T	del	C	A	C	T	C	C	T	T	T	A	T	del	C	TTTTA

Fig. S3. Chromosomal origins of the 323 kb and 244 kb de novo duplications present in Case 1. Genotypes of representative informative SNVs within the duplicated segments are shown, and colour-coded in the proband according to their origin from the mother and father. Note both larger duplicated segments exhibit a paternal origin, but whereas both paternal copies (coloured green and blue) contribute to the 323 kb segment, the 244 kb copies are both sourced from the same (green) paternal chromosome. The SNV within the short (148 bp) duplicated segment (teal) demonstrates that this duplication has a heterologous origin, similar to the 323 kb DUP. Box indicates the T allele present in the abnormal split reads (see Fig. S2).

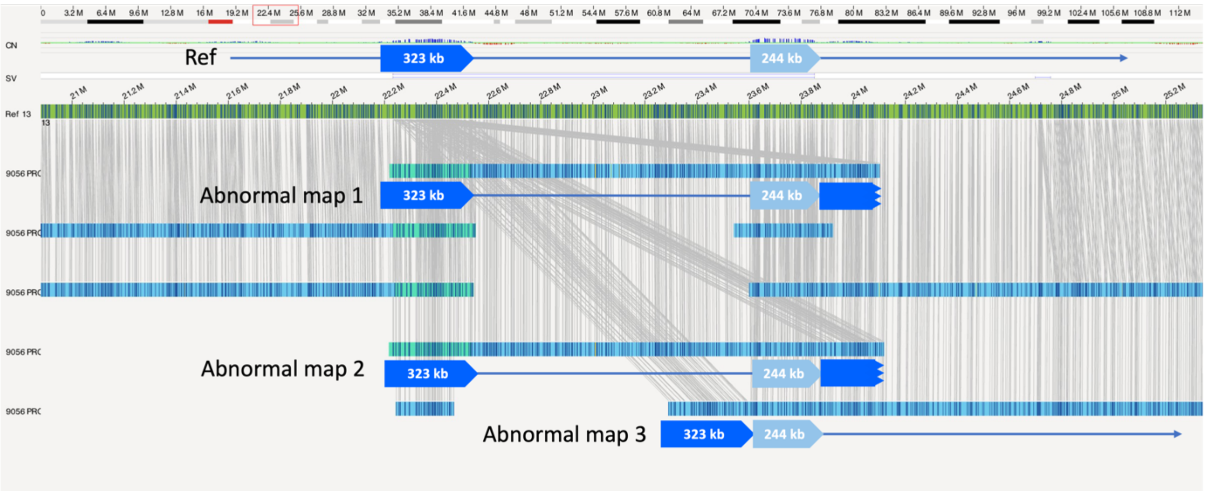

Fig. S4. Initial de novo assembly of OGM data from Case 1 produced by Bionano Access package. Three different abnormal maps were assembled, which appeared to support Alt1 or Alt3 (see Fig. 2A) as the most likely structures.

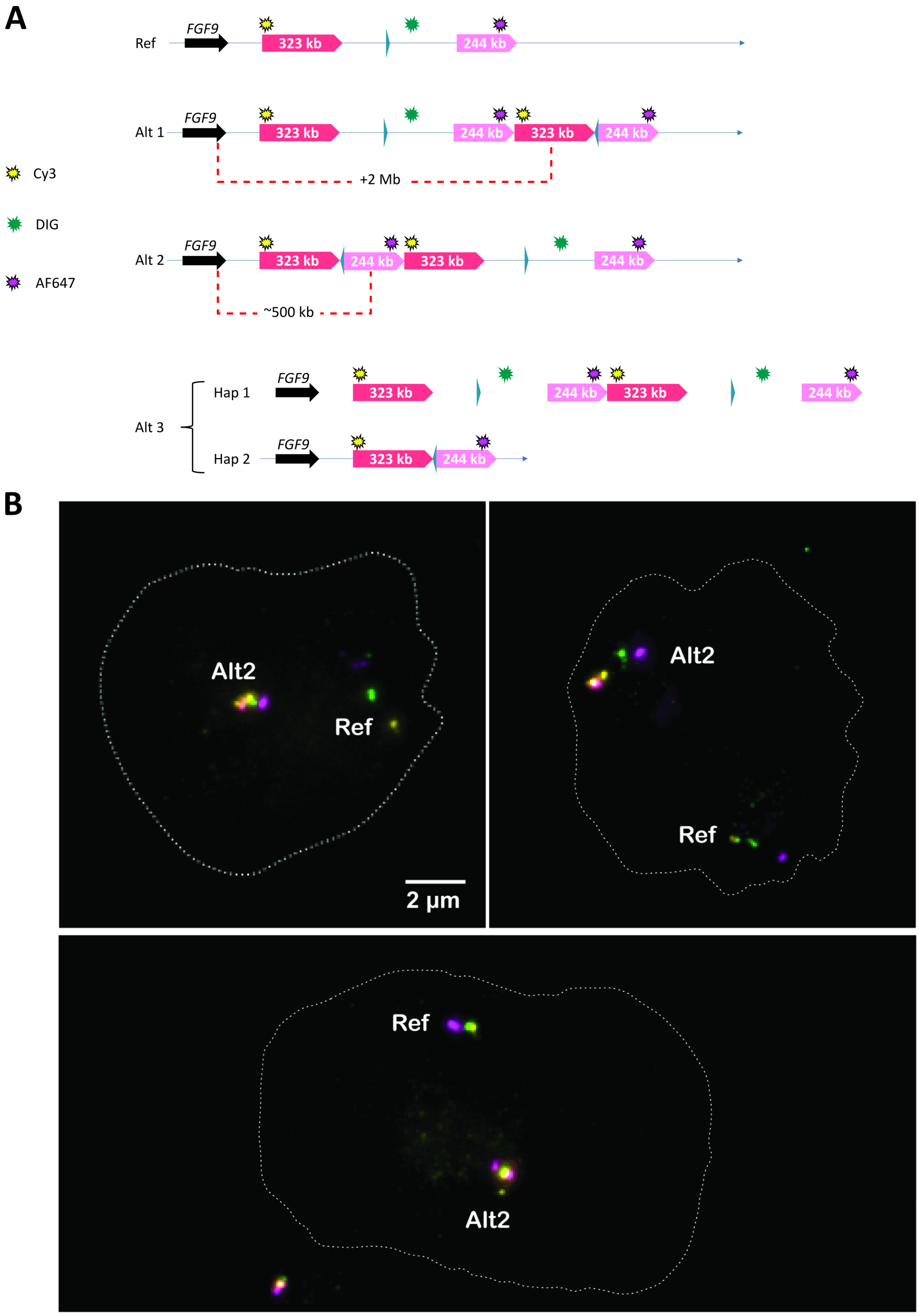

Fig. S5. FISH analysis of patient-derived induced pluripotent stem cells [7] from Case 1. (A) Design showing relative positions of three different probes each labelled with a different fluorophore. (B)

Three representative images of individual cells, showing that one chromosome 13 (Ref) exhibits the expected sequence of all three colours, excluding Alt3, whereas the other chromosome 13 exhibits an abnormal sequence of colours compatible with Alt2 but not Alt1. See page 7 of the Supplementary Material for further information on the analysis of FISH results.

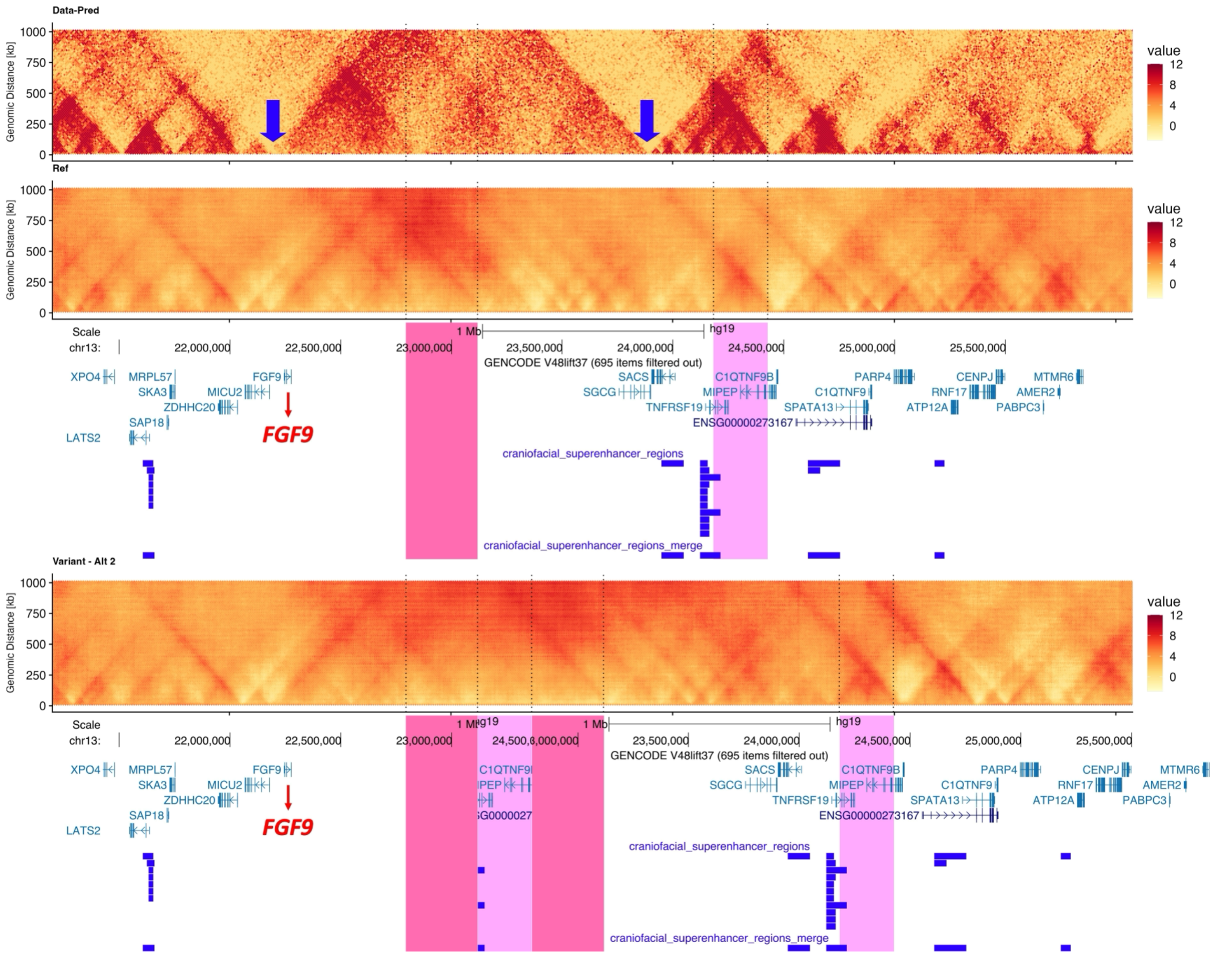

Fig. S6. Observed and predicted TAD structures on chromosome 13. The top panel shows the observed TAD structure based on IMR-90 cell line data [8]. Note large TAD (demarcated by vertical blue arrows), with *FGF9* located at one end. Middle panel, deepC prediction (based on IMR-90 cell line) broadly identifies similar features in the regional TAD structure, including the TAD boundaries. The normal positions of the 323 kb and 244 kb DUP segments are shown as dark and light pink columns, respectively. The lowest panel shows the deepC prediction for the Alt2 configuration, indicating an expansion of the *FGF9*-containing TAD to include the additional 244 kb and 323 kb DUP regions. Genome co-ordinates use the hg19 reference build. Gene tracks generated from UCSC Genome Browser. UCSC session for the reference gene track and craniofacial superenhancer track: https://genome.ucsc.edu/s/429035671/Pei_et_al_2025_figS6_UCSC_track

Case 2 (family ID 347)

Clinical phenotype and additional molecular investigations

The proband is a 16 year old male. There is no family history of neurodevelopmental disorders. He is one of twins conceived via in vitro fertilisation. There were no neurodevelopmental concerns about his sister. He was delivered by elective caesarean section with a birth weight of 2.3 kg (compared to his sister's birth weight of 3.2 kg). He did not require resuscitation. An unusual head shape was noticed at birth as were textured gums. He was noted to have thick head hair and hypertrichosis of his ears and back. Computed tomography imaging of the skull showed early closure of the coronal and sagittal sutures with possible involvement of the other sutures, however, because there was no marked deformity and no evidence of raised intracranial pressure, craniofacial surgery was not performed. He has gingival hypertrophy and has undergone several debulking operations. The hypertrophy has recurred following each procedure. In addition he has hypertrichosis restricted to the external auditory meati. His developmental milestones were at the upper limit of the normal range and he has normal intelligence. Growth parameters are in the normal range.

Array CGH (Nimblegen 135K oligonucleotide array) was reported as showing three copy number gains (reported on hg18 build): arr 16q23.1(75,917,280-77,822,570)x3 dn, arr 17p11.2(18,752,330-19,054,800)x3, arr 17q24.3(65,466,330-65,945,060)x3. The 16q23.1 duplication was reported to have arisen de novo, based on parental qPCR analysis. The 17p11.2 duplication corresponds to a region of known CNV and was not considered pathologically significant. The 17q24.3 duplication was not further investigated. The 16q23.1 and 17q24.3 duplications were classed as being of uncertain clinical significance. In addition, parent-child trio-based exome sequencing was performed (TruSeq exome capture, Bowtie2 alignment, variant calling with SAMtools v1.1 and Platypus v0.5.2; Family 5 reported in Miller et al. [9]), but no pathogenic variant was identified.

A recent G-banded karyotype was normal. As a result of the further molecular investigations described in this report, the final resolved structure was annotated as:

NC_000016.10:g.79267343_qterdelins[NC_000017.11:g.69913550_qter];

NC_000017.11:g.70477257_qterdelins[GTTTAA;NC_000016.10:g.77258535_qter]

A

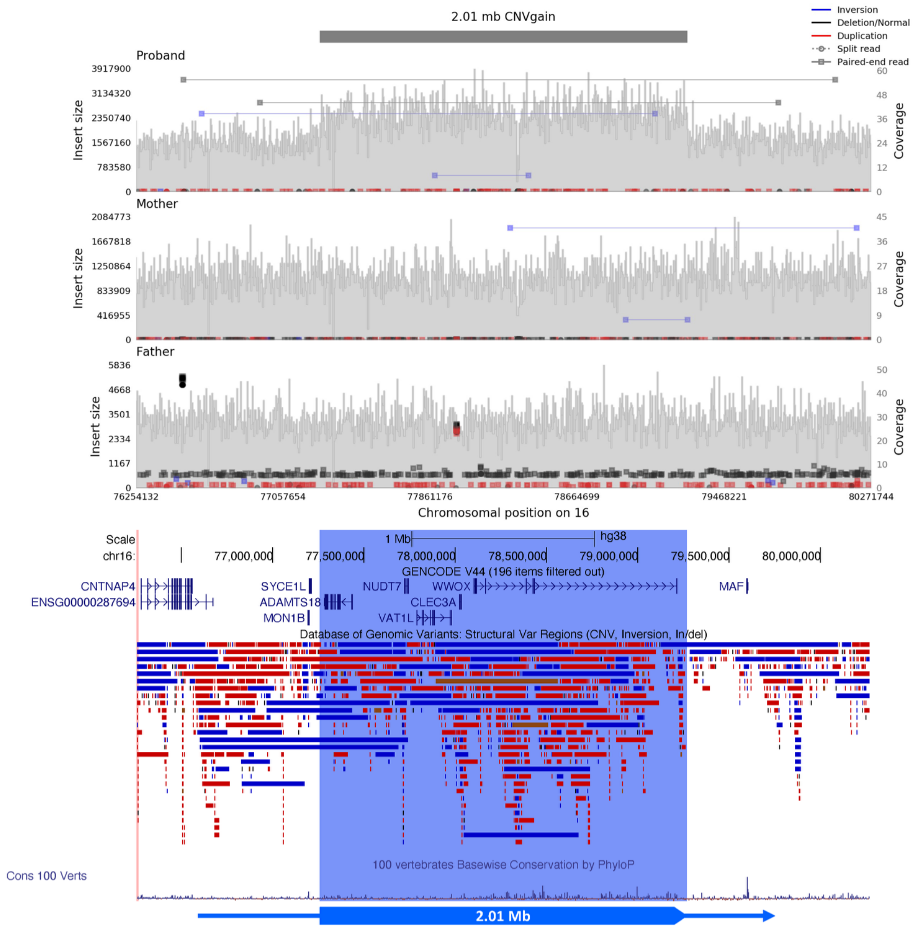
2.01 mb CNVgain
Inversion
Deletion/Normal
Duplication
Split read
Paired-end read
Proband
Mother
Father
Insert size
Coverage
Chromosomal position on 16
76254132
77057654
77861176
78664699
79468221
80271744
Scale
chr16:
1 Mb
hg38
77,000,000
77,500,000
78,000,000
78,500,000
79,000,000
79,500,000
80,000,000
GENCODE V44 (196 items filtered out)
CNTNAP4
ENSG00000287694
SYCE1L
ADAMTS18
MON1B
NUDT7
WWOX
CLEC3A
VAT1L
MAF
Database of Genomic Variants: Structural Var Regions (CNV, Inversion, In/del)
Cons 100 Verts
100 vertebrates Basewise Conservation by PhyloP
2.01 Mb

B

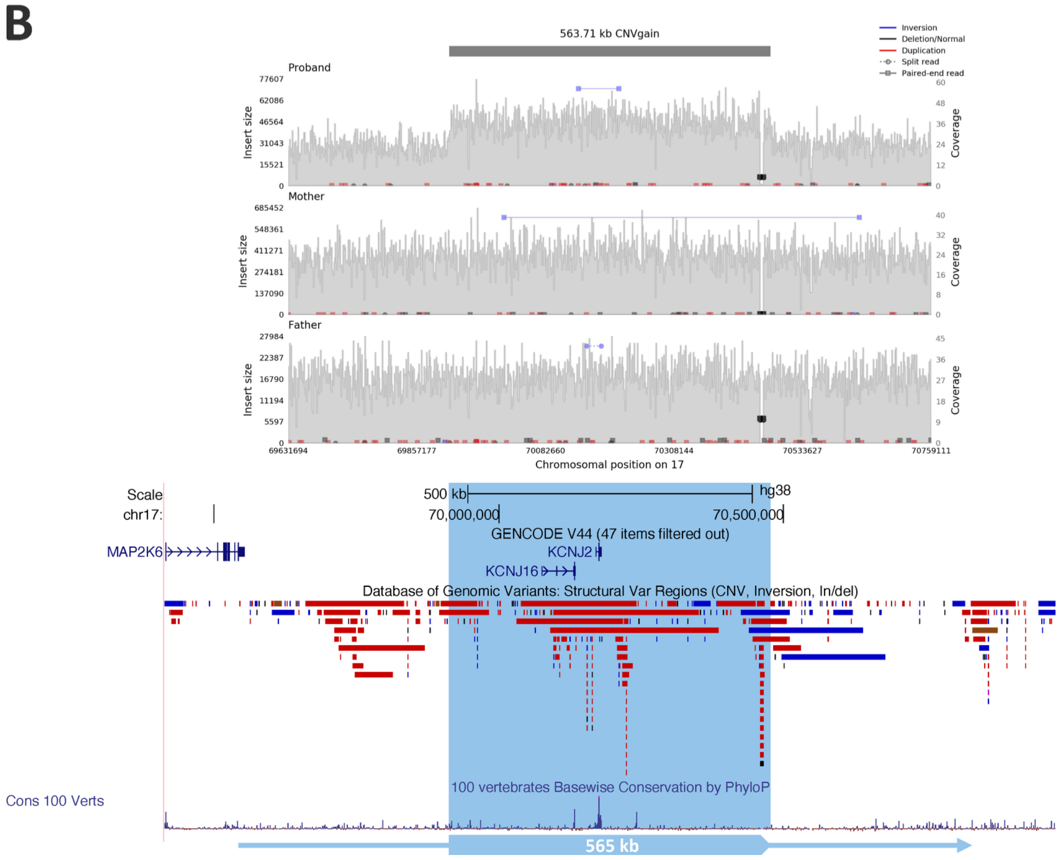
563.71 kb CNVgain
Inversion
Deletion/Normal
Duplication
Split read
Paired-end read
Proband
Mother
Father
Insert size
Coverage
Chromosomal position on 17
69631694
69857177
70082660
70308144
70533627
70759111
Scale
chr17:
500 kb
hg38
70,000,000
70,500,000
GENCODE V44 (47 items filtered out)
MAP2K6
KCNJ2
KCNJ16
Database of Genomic Variants: Structural Var Regions (CNV, Inversion, In/del)
Cons 100 Verts
100 vertebrates Basewise Conservation by PhyloP
565 kb

Fig. S7. Coverage analysis of genome sequence data from Case 2 and parents. (A) Chromosome 16 gain and (B) chromosome 17 gain. Each part is organised in similar fashion to Fig. S1, with (above) Samplot coverage analysis of the proband, mother and father, and (below) annotation of genes, 100-vertebrate sequence conservation, and deletions and duplications from DGV in the UCSC Genome browser. Note that the plots are not shown to identical scales. Both CNV gains have arisen de novo.

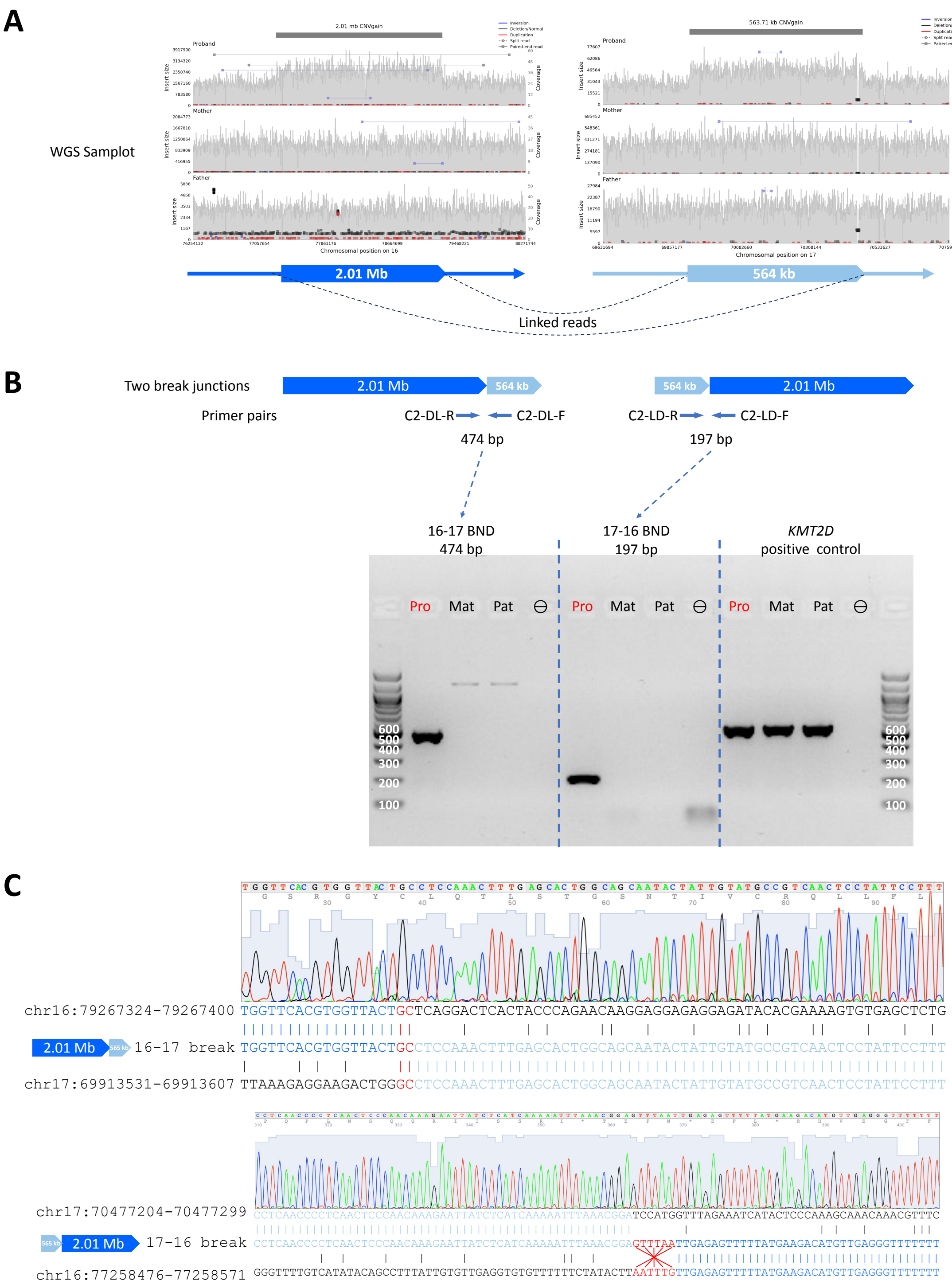

Fig. S8. Characterisation of chromosome 16 and 17 breakpoint junctions in Case 2. (A) Genome sequencing generated paired/split reads linking the two CNV segments together to form two different break junctions. (B) Breakpoint PCR amplified the two break junctions in the proband (Pro) but not the parents (Mat, mother; Pat, father; -, no DNA control); faint bands seen in the parental lanes for the 16-17 amplification represent a non-specific product. (C) Characterisation of the break junctions by dideoxy-sequencing, showing extent of homology at the sequence joins.

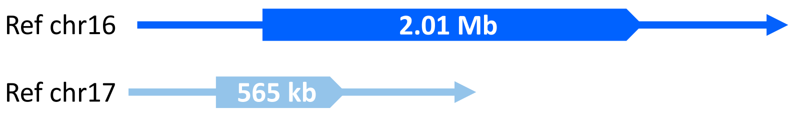

SNP positions on chr16 and chr17 (hg38)	77434463	77735653	79016426	70180084	70067523
Proband	T	T	del	G	GT
	T	T	del	G	GT
	C	C	T	C	G
Mother	T	T	del	G	GT
	C	C	T	C	G
Father	C	C	T	C	G
	C	C	T	C	G

Fig. S9. Chromosomal origins of the 2.01 Mb (chromosome 16) and 565 kb (chromosome 17) de novo duplications present in Case 2. Genotypes of representative informative SNVs/indels within the duplicated segments are shown, and colour-coded in the proband (Pro) according to their origin from the mother (Mat) and father (Pat). Both duplicated segments exhibit a maternal origin, and both show an identical pattern of SNVs/indels indicating they have been sourced from the same maternal chromosome.

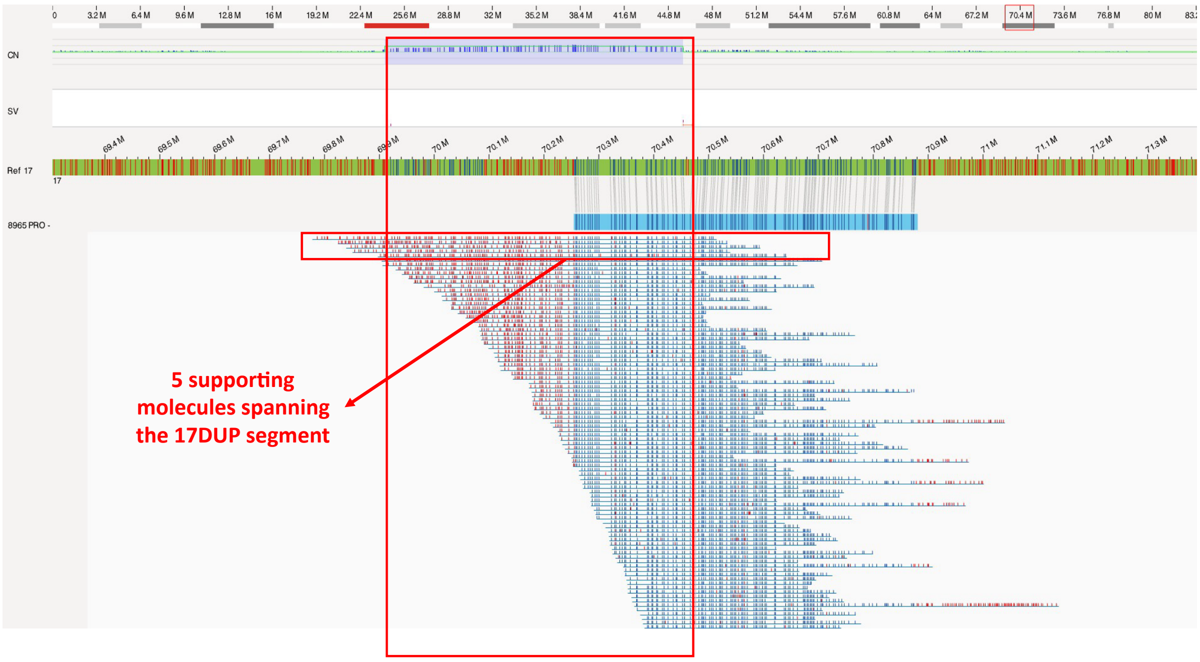

Fig. S10. Use of the Bionano rare variant analysis (RVA) pipeline to identify molecules spanning the DUP17 region in Case 2. The filled blue rectangle is the consensus map aligning to the Ref generated by the native Bionano analysis pipeline. The vertical red box highlights the DUP17 region, which the molecules must span to be informative. The horizontal red box highlights the five potentially informative molecules that appear to span the DUP17 region (see Fig. 3C in the main text for further description).

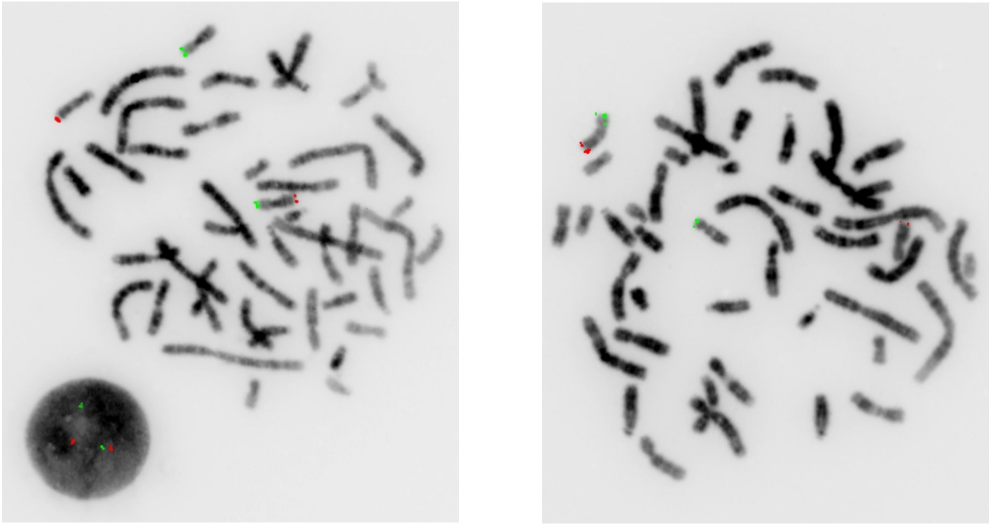

Fig. S11. Additional confirmation of Alt3 (reciprocal translocation) in Case 2 using subtelomeric FISH by a clinical laboratory. Left panel shows result with 16pter (green) and 17qter (red) Cytocell probes. Right panel shows result with 16qter (red) and 17pter (green) probes. Reverse DAPI staining enables chromosomes to be identified by banding pattern; the translocation is cryptic on G-banding.

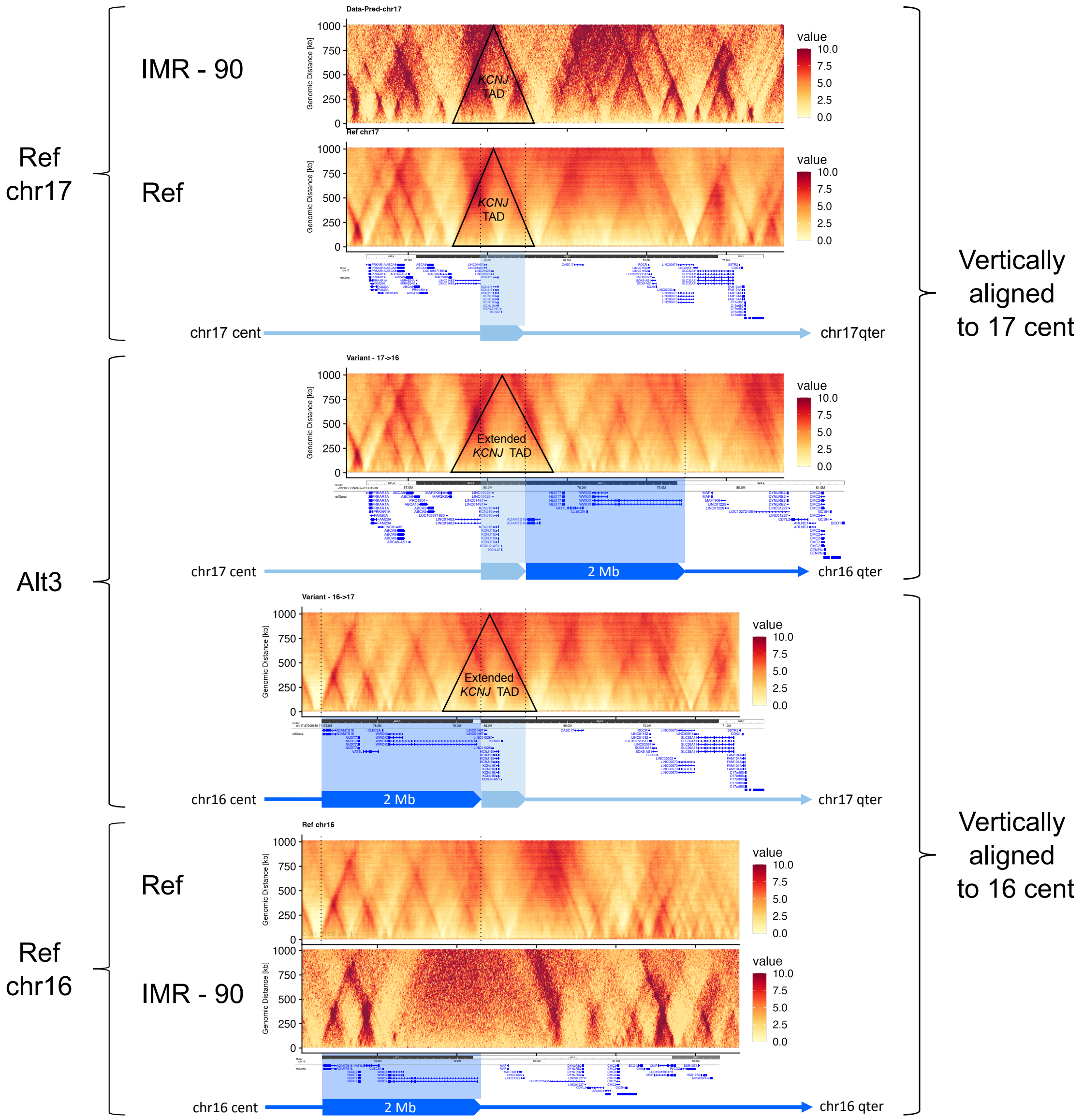

Fig. S12. DeepC TAD prediction for the Alt3 configuration in Case 2. Three groups of tracks are shown for reference chr17, Alt3 configuration, and reference chr16, in descending order. From the top to bottom, the tracks are:

Ref chr17, Hi-C data for the IMR-90 cell line; deepC prediction (“Ref”); RefSeq Genes track on WashU Epigenome Browser; cartoon illustration.

Alt 3 (17cent – 16qter), deepC prediction; RefSeq Genes track on WashU Epigenome Browser; cartoon illustration.

Alt 3 (16cent – 17qter), deepC prediction (“Ref”); RefSeq Genes track on WashU Epigenome Browser; cartoon illustration.

Ref chr16, deepC prediction; Hi-C data for the IMR-90 cell line; RefSeq Genes track on WashU Epigenome Browser; cartoon illustration.

The Ref chr17 IMR-90 panel shows the Hi-C data for the IMR-90 cell line [8] aligned to genes in the WashU Epigenome Browser (hg19 build), demonstrating that the DUP17 (pale blue rectangle) contains two genes, *KCNJ16* and *KCNJ2*, and lies within a TAD ("*KCNJ* TAD") delineated by the black triangle, which is separate both from a centromeric TAD including members of the *ABCA* gene family, and a telomeric TAD containing the large *SOX9* regulatory region. Below, the Ref panel depicts the corresponding deepC prediction for this region, which shows a similar TAD structure to that observed experimentally. The middle Alt 3 panels show the deepC predictions for each of the abnormal chromosomes that constitute the Alt3 topology. Note the widening of the TAD boundaries in each derivative chromosome to include ectopic chromosome 16 sequence from the DUP16 (dark blue rectangles) within the respective neo-TADs (black triangles). The lower Ref chr16 panels show the Hi-C data for the IMR-90 cell line as well the corresponding deepC prediction (Ref) for the reference chr16 region. Gene tracks generated from WashU Epigenome Browser [10].

Case 3 (family ID 302)

Clinical phenotyping and additional molecular investigations

The female proband is the only child born to unrelated parents of north European origin. She has a half-sister on her mother's side who is well. Neither parent has any medical problems nor any obvious craniofacial abnormalities. There is no other relevant family history. The pregnancy was complicated by pre-eclampsia and decreased fetal movements from 26 weeks. She was born at 31 weeks' gestation by emergency Caesarean section due to maternal eclampsia. The birth weight was 1190 g and she required respiratory support for the first week of life. At birth, an irregular head shape was noted suggestive of pansynostosis. She was referred to the local craniofacial unit and underwent surgical correction at the ages of 10 days and again at 2 months, when she had calvarial remodelling and forehead advancement. Clinical genetics review at the age of 2 years revealed a crouzonoid facial appearance and mild developmental delay, but no other abnormalities.

Genetic tests with normal results included sequencing of *FGFR1*, *-2*, *-3* and *TWIST1*, and a G-banded karyotype. Array CGH (BlueGnome 8x60k v2.0 ISCA platform) was reported as arr 20p12.3p12.2(9,156,082-9,697,403)x3 mat [hg19]; subsequent targeted microarray analysis had revealed the same rearrangement in the mother. As the mother has a normal phenotype, the chromosome 20 rearrangement was thought likely to be coincidental to Case 3's clinical features. As part of the investigations described in this work, a blood sample was obtained from the healthy maternal grandmother who, based on breakpoint PCR analysis, also carried the rearrangement. Additionally, parent-child trio-based exome sequencing was performed (TruSeq exome capture, Bowtie2 alignment, variant calling with SAMtools v1.1 and Platypus v0.5.2; Family 2 reported in Miller et al. [9]), but no pathogenic variant was identified. The family was therefore referred for genome sequencing as part of the 100kGP.

Based on the dideoxy-sequencing results (Fig. S15) combined with OGM (Fig. 4), the likely sequence structure for the mother (Alt 5 topology) was annotated to be:

NC_000020.11:g.12319713_12319714ins[9153480_9780418inv;12350710_12657715inv;12564056_12626497inv;12115100_12319713].

Table S7: Abnormal Canvas and Manta calls located on chromosome 20 from genome sequencing data of Case 3.

CHROM	POS	END	SVTYPE	SVLEN	ID	QUAL
**chr20**	12114875	12319079	CNV	.	Canvas:GAIN:chr20:12114876:12319079	30
**chr20**	12115099	12564055	INV	448956	MantaINV:170090:0:1:0:0:0	516
**chr20**	12115099	12564055	INV	448956	MantaINV:148874:0:1:0:0:0	462
**chr20**	12318903	12350589	.	.	Canvas:REF:chr20:12318903:12350589	10
**chr20**	12319080	12350931	.	.	Canvas:REF:chr20:12319080:12350931	11
**chr20**	12350589	12564071	CNV	.	Canvas:GAIN:chr20:12350590:12564071	28
**chr20**	12350709	12626497	DUP	275788	MantaDUP:TANDEM:170096:0:1:0:0:0	616
**chr20**	12350709	12626497	DUP	275788	MantaDUP:TANDEM:148880:0:1:0:0:0	394

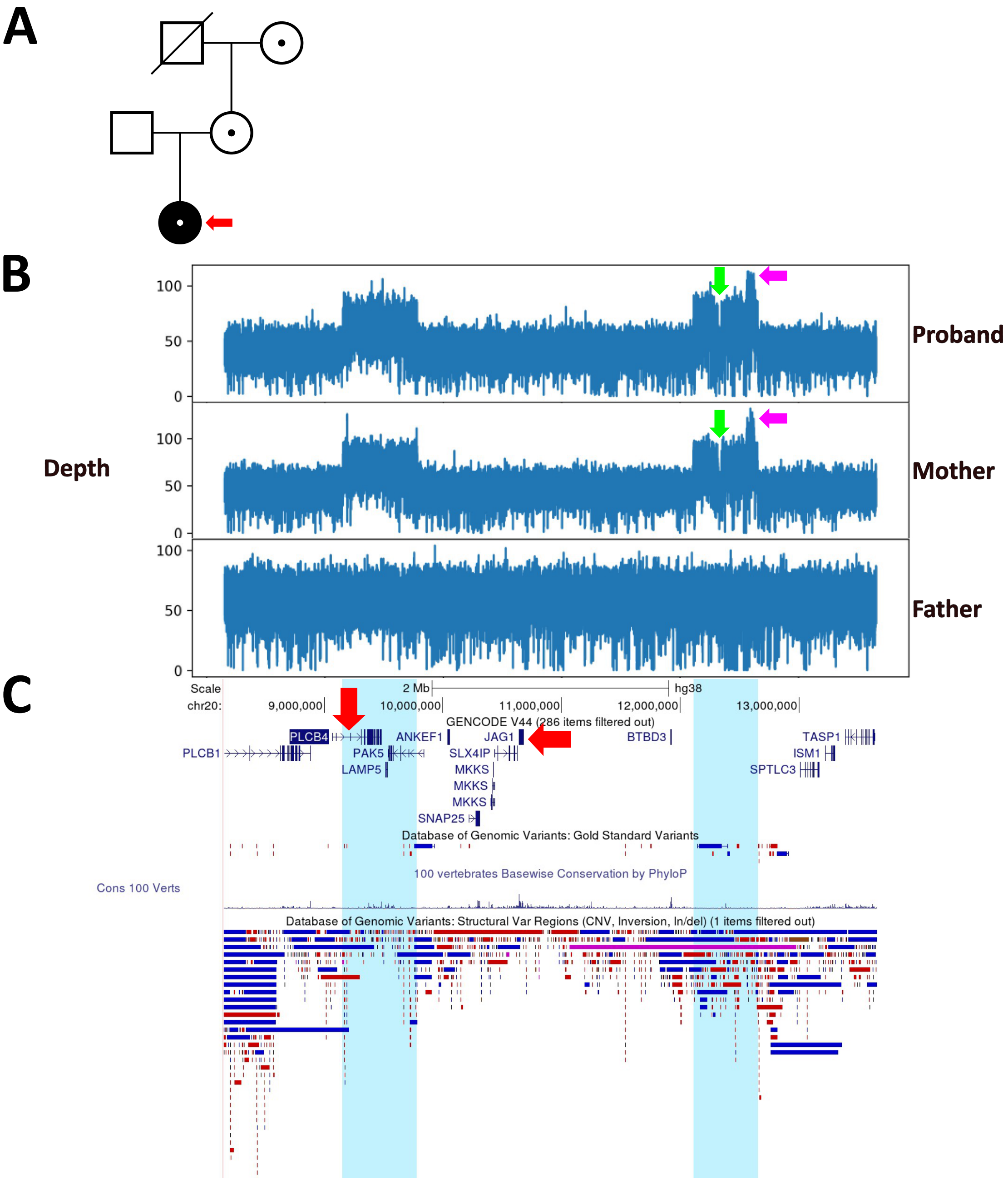

Fig. S13. Genome sequencing of Case 3 reveals multiple regions of copy number gain of chromosome 20. (*A*) Simplified pedigree showing the family members analysed. The filled symbol denotes the affected proband, central dots indicate heterozygotes for the chromosome 20 rearrangement. (*B*) Sequence depth plot of relevant section of chromosome 20. Note two separate regions with increased copy number in proband and mother; in the more centromeric region to the right, additional complexity is evident with subregions of normal (green arrows), or further increased copy number (pink arrows). (*C*) Genetic context of the SV viewed in UCSC Genome Browser, with possible candidate genes, *PLCB4* and *JAG1*, highlighted by red arrows. Annotations follow the same order as in Figs S1 and S7.

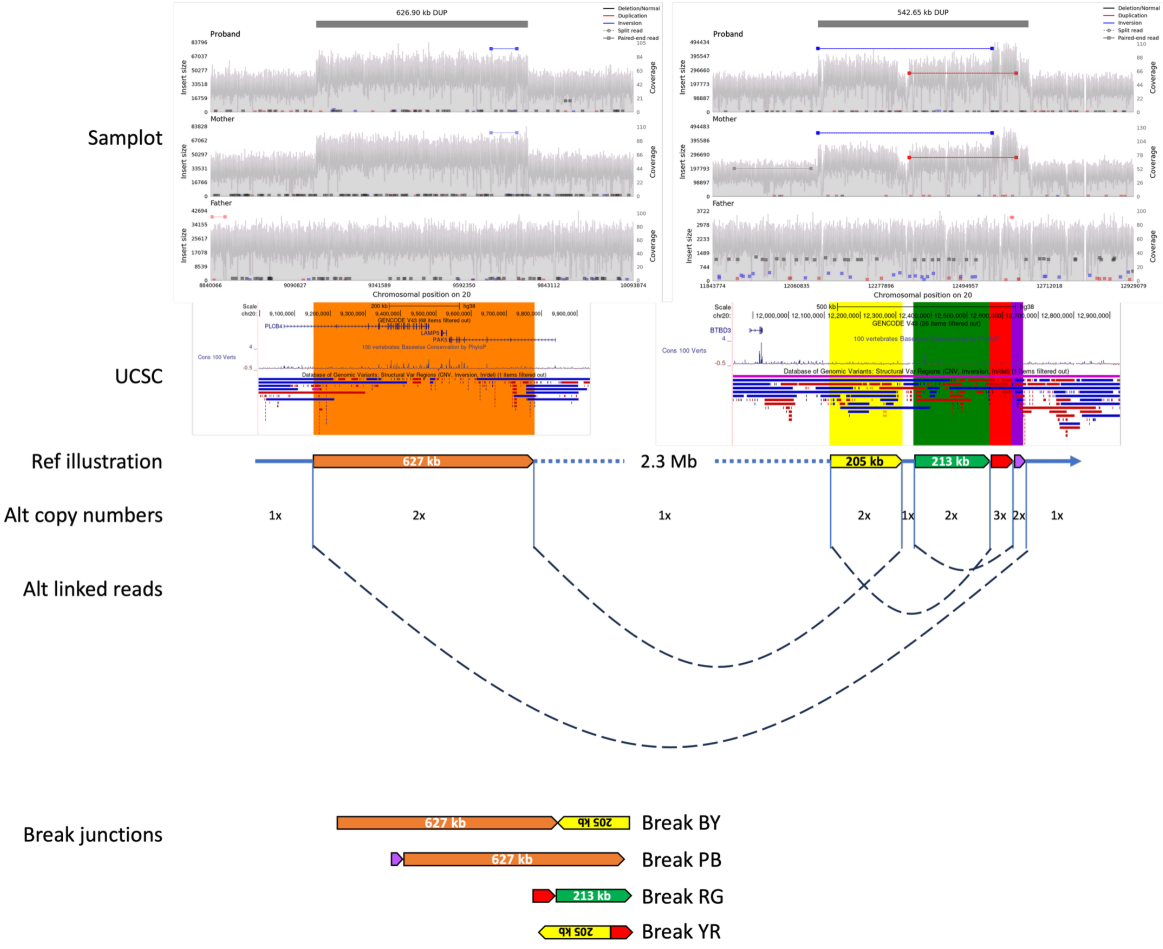

Fig. S14. Analysis of genome sequence to show connectivity of breakpoints in Case 3. From the Samplot coverage and linked reads information, five segments of abnormal copy number (CN) are illustrated using the following colour scheme: Brown [B] (2x chr20:9153480-9780418), Yellow [Y] (2x chr20:12115100-12319713), Green [G] (2x chr20:12350710-12564055), Red [R] (3x chr20:12564056-12626497), and Purple [P] (2x chr20:12626498-12657715).

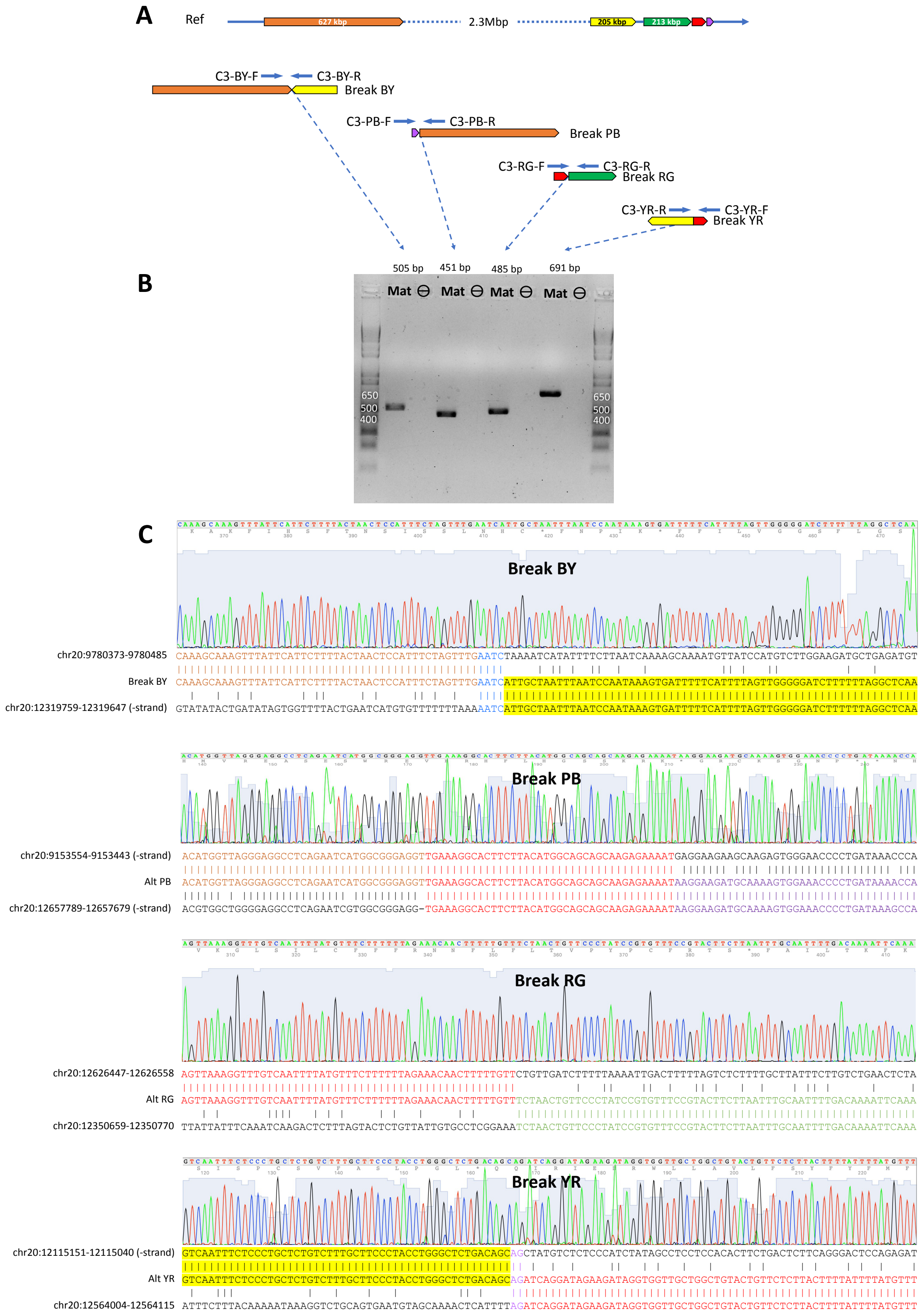

Fig. S15. Breakpoint PCR and dideoxy-sequencing verification of the four breaks in the Case 3 family originally detected by genome sequencing. (A) Cartoon showing the structure of the reference locus

(Ref) and primers used to amplify the four break junctions. (B) Breakpoint PCR result showing that all four breaks can be amplified in the maternal DNA sample (similar results were obtained for DNA samples from the proband and maternal grandmother; not illustrated). (C) Dideoxy-sequencing characterised in detail all four amplified breaks.

Ref

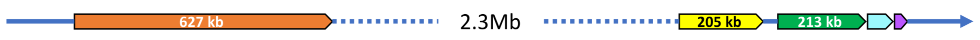

SNP positions on chr20 (hg38)		9162894	9198026	9245737	9398301	9630801	12117971	12129541	12144601	12159408	12363347	12565627	12566231	12566367	12568278	12568869	12571586	12627254	12657682
Proband		C	C	G	A	G	del	G	T	G	A	T	A	T	T	C	T	A	C
		C	C	G	A	G	del	G	T	G	T	T<br>T	A<br>A	T<br>T	A<br>A	T<br>T	T<br>T	G	T
		G	T	GT	T	A	A	A	C	A	T	C	G	C	T	T	C	G	T
Mother		C	C	G	A	G	A	A	C	G	A	T	A	T	T	T	T	A	C
		C	C	G	A	G	del	G	T	G	T	T<br>T	A<br>A	T<br>T	A<br>A	T<br>T	T<br>T	G	T
		G	T	GT	T	A	A	A	C	A	T	C	G	C	T	T	C	G	T
Father		C	C	G	A	G	del	G	T	G	A	T	A	T	T	C	T	A	C
		C	C	G	A	G	del	G	T	G	A	T	A	T	A	T	T	A	C

Fig. S16. Chromosomal origins of the four duplicated, and one triplicated segments involved in the inherited chromosome 20 rearrangement present in Case 3 and her mother. Genotypes of representative informative SNVs/indels within each of the duplicated segments are shown, and colour-coded in the proband (Pro) according to their origin from the mother (Mat) and father (Pat). In three duplicated segments (brown, yellow and red), multiple markers showed transmission of two different SNV/indels alleles from mother to child, demonstrating that the duplicated/triplicated locus had a heterologous origin. The other two duplicated regions did not show this pattern, consistent with, but not proving, that the duplicated sequence originally arose from the homologous chromosome.

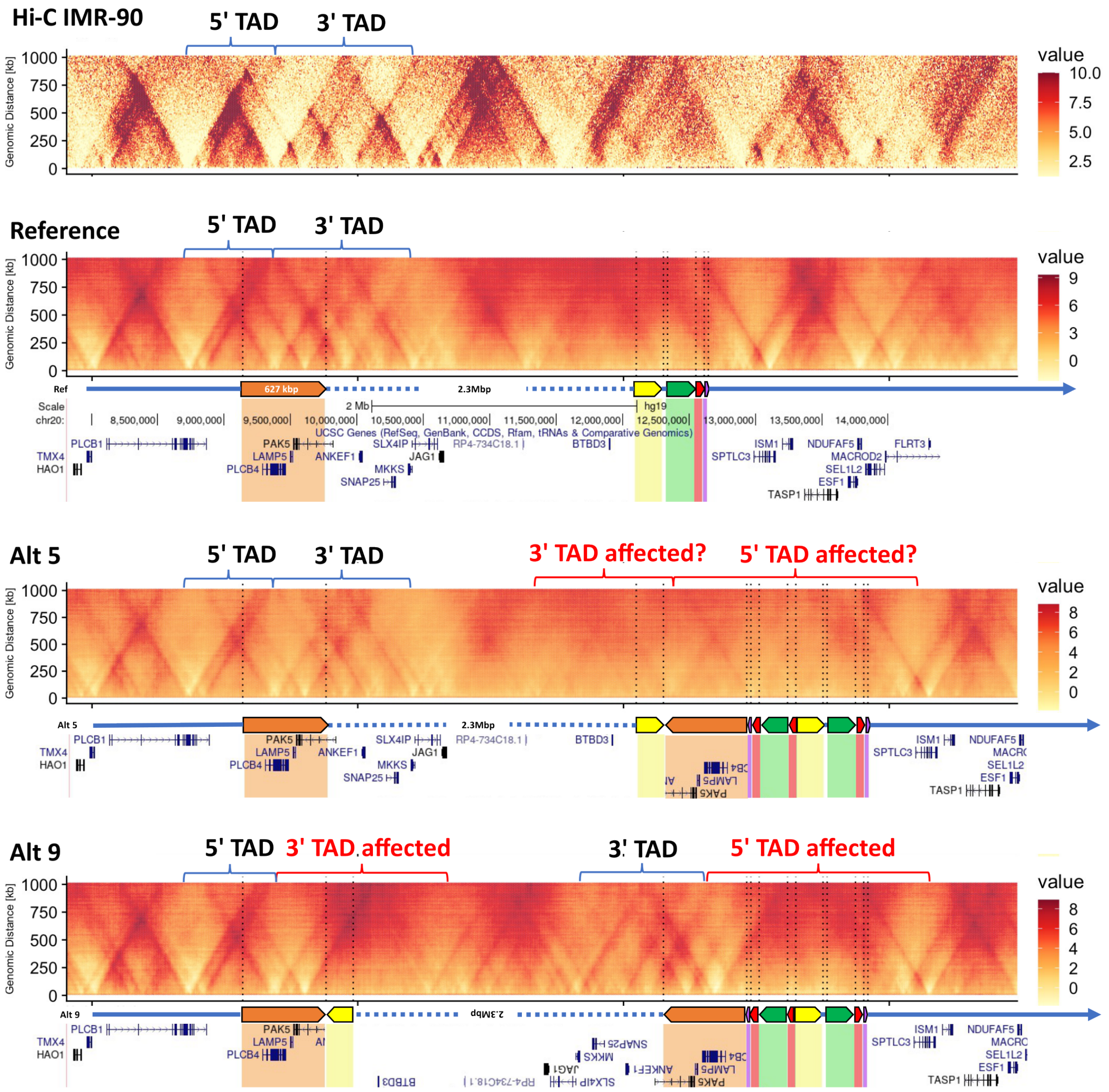

Fig. S17. DeepC predictions for the two alternative topologies, Alt5 and Alt9, potentially present in the Case 3 cxSV. In the Hi-C data track (IMR-90 cell line), a TAD boundary appears to coincide with *PLCB4*, suggesting that this gene could be regulated within either or both TADs – the 5' TAD and the 3' TAD, marked in blue brackets. The corresponding deep-C reference prediction shows good concordance for the TAD structures around *PLCB4*. The two Alt topologies exhibit marked differences in predicted effect on TAD structure around the *PLCB4* homologs. In Alt5, only the centromeric homolog lies in an abnormal sequence context and no clear TAD structure is evident. By contrast, in Alt9, both *PLCB4* copies lie in abnormal sequence contexts and clearer alterations in TAD structure are predicted. Likely altered TADs are marked in red brackets. Note also that according to the IMR-90 Hi-C data *JAG1*, the second gene considered a potential candidate, lies deep within the intervening single copy sequence that includes its entire TAD, so dysregulation appears unlikely with

either the Alt5 or Alt9 rearrangements. Schematics and gene track colour scheme follow the previous figures. Figure in hg19.

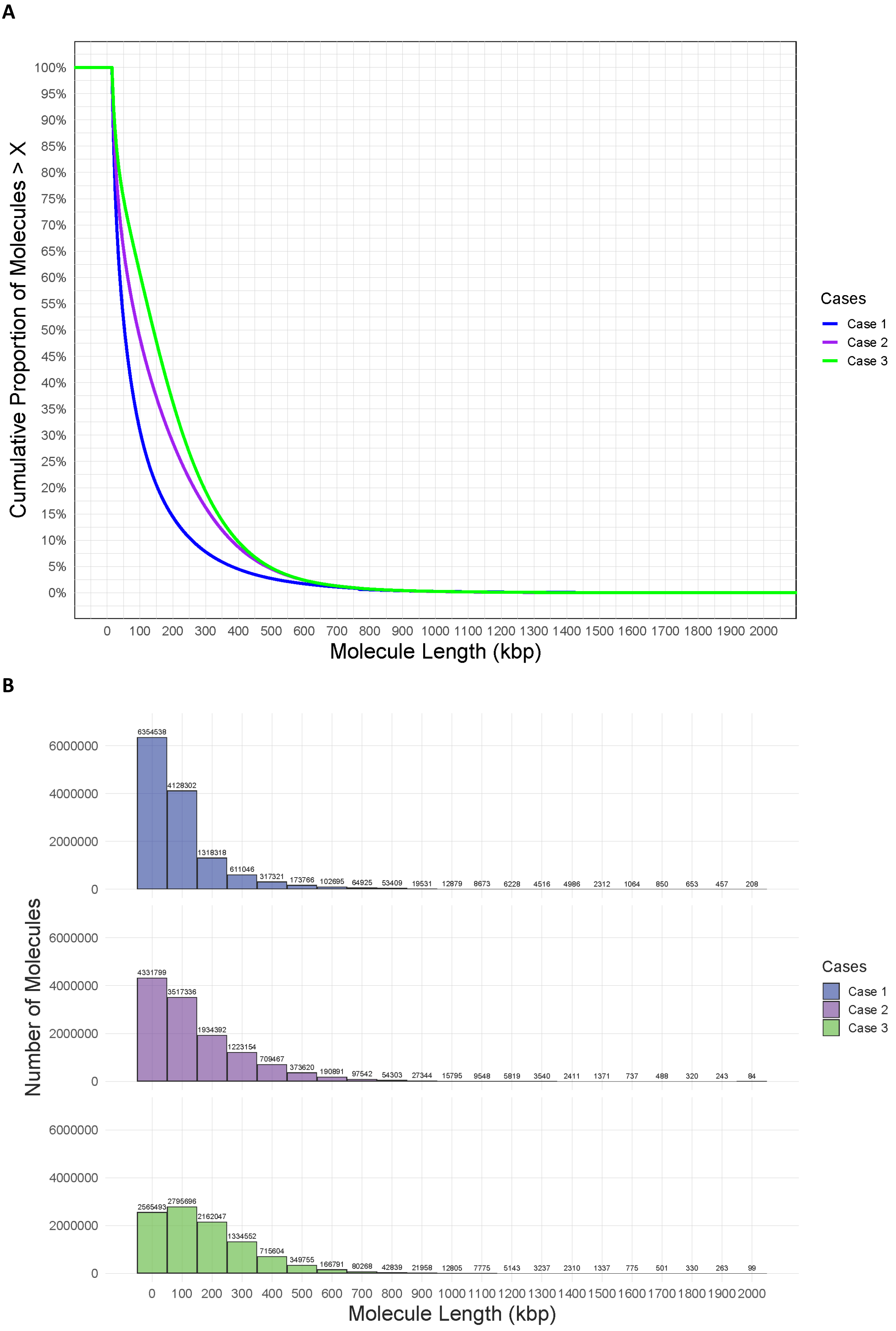

Fig. S18. Size distribution (A) and absolute number (B) of molecules analysed in Cases 1-3.

Supplementary References

1. Riggs ER, Andersen EF, Cherry AM, Kantarci S, Kearney H, Patel A, et al. Technical standards for the interpretation and reporting of constitutional copy-number variants: a joint consensus recommendation of the American College of Medical Genetics and Genomics (ACMG) and the Clinical Genome Resource (ClinGen). Genet Med. 2020;22(2):245-57.

2. Ellingford JM, Ahn JW, Bagnall RD, Baralle D, Barton S, Campbell C, et al. Recommendations for clinical interpretation of variants found in non-coding regions of the genome. Genome Med. 2022;14(1):73.

3. Richards S, Aziz N, Bale S, Bick D, Das S, Gastier-Foster J, et al. Standards and guidelines for the interpretation of sequence variants: a joint consensus recommendation of the American College of Medical Genetics and Genomics and the Association for Molecular Pathology. Genet Med. 2015;17(5):405-24.

4. Collins RL, Glessner JT, Porcu E, Lepamets M, Brandon R, Lauricella C, et al. A cross-disorder dosage sensitivity map of the human genome. Cell. 2022;185(16):3041-55.

5. Morison LD, Meffert E, Stampfer M, Steiner-Wilke I, Vollmer B, Schulze K, et al. In-depth characterisation of a cohort of individuals with missense and loss-of-function variants disrupting *FOXP2*. J Med Genet. 2023;60(6):597-607.

6. Tonne E, Due-Tonnessen BJ, Vigeland MD, Amundsen SS, Ribarska T, Asten PM, et al. Whole-exome sequencing in syndromic craniosynostosis increases diagnostic yield and identifies candidate genes in osteogenic signaling pathways. Am J Med Genet A. 2022;188(5):1464-75.

7. Korona D, Hashimoto, A.S., Pei, Y., Calpena, E., Sloane-Stanley, J., Riva, S.G., Schwessinger, R., Forzano, F., Chintawar, S., Duggal, G., Wall, S.A., Hughes, J.R., Twigg, S.R.F., Wilkie, A.O.M. Evaluating the pathogenic significance of unique chromosomal variants in craniosynostosis using patient-derived induced pluripotent stem cells and mouse modelling. Submitted for publication. 2025.

8. Rao SSP, Huntley MH, Durand NC, Stamenova EK, Bochkov ID, Robinson JT, et al. A 3D Map of the Human Genome at Kilobase Resolution Reveals Principles of Chromatin Looping. Cell. 2014;159(7):1665-80.

9. Miller KA, Twigg SRF, McGowan SJ, Phipps JM, Fenwick AL, Johnson D, et al. Diagnostic value of exome and whole genome sequencing in craniosynostosis. J Med Genet. 2017;54(4):260-8.

10. Li DF, Purushotham D, Harrison JK, Hsu S, Zhuo XY, Fan CX, et al. WashU Epigenome Browser update 2022. Nucleic Acids Res. 2022;50(W1):W774-W81.
